# Artificial Diets with Selective Restriction of Amino Acids and Very Low Levels of Lipids Induce Anticancer Activity in Mice with Metastatic Triple-Negative Breast Cancer

**DOI:** 10.3390/cancers15051540

**Published:** 2023-02-28

**Authors:** Emilio Guillén-Mancina, Julio José Jiménez-Alonso, José Manuel Calderón-Montaño, Víctor Jiménez-González, Patricia Díaz-Ortega, Estefanía Burgos-Morón, Miguel López-Lázaro

**Affiliations:** Department of Pharmacology, Faculty of Pharmacy, University of Seville, 41012 Sevilla, Spain

**Keywords:** amino acids, lipids, diet, low-fat diet, cancer therapy, cancer metabolism, triple-negative breast cancer, lung cancer, melanoma, colorectal cancer, ovarian cancer, metastasis

## Abstract

**Simple Summary:**

Current treatments for patients with metastatic triple negative breast cancer (TNBC) are generally ineffective. This manuscript shows for the first time that the survival of mice with metastatic TNBC can be markedly increased through dietary manipulation. Our study revealed that the survival of some mice with metastatic TNBC was increased by replacing their normal diet with artificial diets in which the levels of amino acids (AAs) are manipulated, and the levels of lipids are markedly reduced. The anticancer activity of this non-pharmacological strategy was higher than that of drugs currently used in the treatment of patients with metastatic TNBC. This anticancer strategy also increased the survival of mice with other types of metastatic cancers. Manipulation of AA and lipid levels with artificial diets may be a useful strategy to treat patients with metastatic TNBC and other types of disseminated cancer.

**Abstract:**

Patients with metastatic triple negative breast cancer (TNBC) need new therapies to improve the low survival rates achieved with standard treatments. In this work, we show for the first time that the survival of mice with metastatic TNBC can be markedly increased by replacing their normal diet with artificial diets in which the levels of amino acids (AAs) and lipids are strongly manipulated. After observing selective anticancer activity in vitro, we prepared five artificial diets and evaluated their anticancer activity in a challenging model of metastatic TNBC. The model was established by injecting 4T1 murine TNBC cells into the tail vein of immunocompetent BALB/cAnNRj mice. First-line drugs doxorubicin and capecitabine were also tested in this model. AA manipulation led to modest improvements in mice survival when the levels of lipids were normal. Reducing lipid levels to 1% markedly improved the activity of several diets with different AA content. Some mice fed the artificial diets as monotherapy lived much longer than mice treated with doxorubicin and capecitabine. An artificial diet without 10 non-essential AAs, with reduced levels of essential AAs, and with 1% lipids improved the survival not only of mice with TNBC but also of mice with other types of metastatic cancers.

## 1. Introduction

Breast cancer is the most common malignancy in women [1,2]. Triple negative breast cancer (TNBC) is a subtype of breast cancer defined by negative expression of estrogen receptor, progesterone receptor and human epidermal growth factor receptor-2. It represents approximately 15–20% of all breast cancer cases [3]. TNBCs generally have an aggressive behavior and treatment options are more limited than for other subtypes of breast cancer. The 5-year survival rate of women diagnosed with metastatic TNBC is approximately 12% [2].

Pharmacotherapy is the main form of treatment for patients with metastatic TNBC. The preferred treatment options for patients with stage IV TNBC are the anthracyclines doxorubicin or liposomal doxorubicin, the antimetabolites capecitabine or gemcitabine, the microtubule inhibitors paclitaxel, vinorelbine or eribulin, and the antibody–drug conjugate sacituzumab govitecan [4]. Alternative first-line treatments can be used when certain biomarkers are detected. When *BRCA* mutations are identified, PARP inhibitors (olaparib) or platinum compounds (cisplatin or carboplatin) can be used [4,5,6]. For PD-L1 expressing TNBC, the preferred treatment option is pembrolizumab plus a chemotherapy drug [4,7]. Although these drugs can increase patient survival and palliate disease-related symptoms, they do not usually cure the disease. New therapies for patients with metastatic TNBC are therefore highly needed.

TNBC cells acquire metabolic alterations [8,9,10] that may be exploited to develop new therapies for patients with metastatic TNBC. Cancer cells reprogram their metabolism to fulfill their elevated energy demands, to produce the large amounts of building blocks required for biosynthesis and proliferation, and to survive under conditions of elevated oxidative stress [11]. Cancer cells commonly develop alterations in amino acid (AA) metabolism. For example, many cancer cells rely on external sources of non-essential AAs (NEAAs) to maintain their proliferative demands and redox homeostasis [11,12]. Targeting the altered AA metabolism of cancer cells using pharmacological and dietary strategies shows promising results against a wide variety of cancers [11,12]. In breast cancer, pharmacological strategies have been developed to target cystine (CySS) uptake [13,14], to disrupt glutamine (Gln) catabolism [15], to inhibit proline (Pro) catabolism [16,17], and to deplete plasma levels of asparagine (Asn), arginine (Arg) and cyst(e)ine (Cys). The enzymes L-asparaginase [18,19,20], ADI-PEG20 [21] and cyst(e)inase [22] have shown anticancer activity in murine breast cancer models, and ADI-PEG20 in combination with doxorubicin has recently been evaluated in a phase I clinical trial in patients with metastatic breast cancer and other metastatic tumors [23]. Dietary restriction of several AAs has also shown in vivo anticancer effects in TNBC models, including restrictions of methionine (Met) [24,25,26,27], Arg [28], Asn [19,20], and double restriction of serine (Ser) and glycine (Gly) [29].

Cancer cells develop changes in lipid metabolism that may also be exploited to develop new therapies for patients with metastatic TNBC. Dietary lipids provide high levels of fatty acids. Cancer cells use these fatty acids to fulfill their energy demands, to produce lipid bilayers for the new cells created during tumor growth, and to support many other processes involved in cancer survival, progression and metastasis [30,31]. For example, dietary lipids provide linoleic acid. This omega-6 essential fatty acid is a precursor of arachidonic acid, which cancer cells avidly consume to generate pro-inflammatory prostaglandins and leukotrienes that have crucial roles in many processes involved in cancer progression [32]. Some cancer cells are also known to overexpress CD36, a membrane protein used for the cellular uptake of fatty acids [33]. The overexpression of this membrane protein is correlated with a poor prognosis of breast cancer and other types of cancer, and its pharmacological inhibition decreases the metastatic potential in murine cancer models, including TNBC models [33]. Since lipid availability is crucial for cancer development, it is not surprising that high-fat diets accelerate cancer progression in mice [34,35,36,37,38,39,40,41,42,43,44], including mice with TNBC and other subtypes of breast cancer [45,46,47,48]. Clinical data have also shown that the levels of dietary lipids influence disease progression in women with breast cancer. Recurrence of early-stage resected breast cancer was higher in women with a high-fat diet compared to women with a low-fat diet [49]. A low-fat dietary pattern was also found to reduce mortality in women after breast cancer [50]. It is important to note that low-fat diets in these preclinical and clinical studies consisted of diets with 5–10% of lipids in their composition; the anticancer effects of diets with a percentage of lipids below 5% is underexplored.

We have recently shown that artificial diets based on selective amino acid restriction induce marked anticancer activity in mice with colon and renal cancers [51,52]. In this article, after observing that an artificial media lacking 10 NEAAs induced selective anticancer activity in TNBC cells in vitro, we show that a diet lacking these 10 AAs induced anticancer activity in a challenging animal model of metastatic TNBC. The in vivo anticancer activity of this diet was markedly improved when the lipid levels were reduced from 14% to 1%. Four artificial diets with selective AA restrictions and very low levels of lipids (1%) induced anticancer activity in mice with metastatic TNBC. Mean survivals in mice fed these diets were higher than in mice treated with the standard therapies doxorubicin or capecitabine. One of the diets also induced anticancer activity in mice with other types of metastatic cancers. Although none of the animals used in this work were cured by any standard or experimental treatment, several mice treated with our artificial diets had very long survivals.

## 2. Materials and Methods

### 2.1. Drugs and Reagents

MTT (3-(4,5-dimethylthiazol-2-yl)-2,5-diphenyltetrazolium bromide, A22310005) and SDS (sodium dodecyl sulfate, 1423631209) were obtained from Panreac Applichem (Darmstadt, Germany). 5-fluorouracil (5-FU) and resazurin were purchased from Sigma (Kawasaki, Japan). Cisplatin, choline bitartrate (450225000), tert-butylhydroquinone (TBHQ, 150822500) and casein (isolated from bovine milk, 276070010) were purchased from Thermo Scientific Acros Organics (Waltham, MA, USA). We also used doxorubicin (50 mg powder for solution, Farmiblastina, Pfizer (New York, NY, USA), 958314.9), capecitabine (500 mg/tablet, Sandoz, Basel, Switzerland), anti-PD-1 (anti-mouse PD-1 (CD279), clone RMP1-14, BE0146, Bioxcell, Lebanon, NH, USA), India Ink (Superblack India Ink, Speedball (Statesville, NC, USA), 33X089A), and sterile physiological serum for diluting injected drugs (Kin laboratory, Dos Hermanas, Spain, 160407.1). Mineral Mix (AIN-93M-MX, 960401) and Vitamin Mix (AIN Vitamin Mixture 76, 905454) were purchased from MP Biomedicals (Eschwege, Germany). Sucrose was obtained from a local market (MAS Supermarket, Seville, Spain). Cellulose and corn starch were purchased from Farmusal (local pharmacy, Granada, Spain). Extra virgin olive oil (marketable olive oil developed for Dia Supermarket, Spain, 112529), salmon oil (marketable oil developed for Pets Purest, England, B06WWFTRXM) and coconut oil (marketable oil developed for Mercadona Supermarket, Spain, 848000041937) were used as a fat source. Essential amino acid mix and L-glutamine (Gln) were obtained from Myprotein (Manchester, England).

L-alanine (Ala, A1688), L-arginine (Arg, A3675), L-asparagine-1-hydrate (Asn, A1668), L-aspartic acid (Asp, A3715), L-cysteine (Cys, A3694), L-cystine (CySS, A1703), L-glutamic acid (Glu, A1704), glycine (Gly, A3707), L-leucine (Leu, A1426), L-methionine (Met, A1340), L-proline (Pro, A1707), L-serine (Ser, A1708) and L-tyrosine (Tyr, A3437) were purchased from Panreac Applichem. Cell culture reagents were obtained from Biowest (Nuaillé, France) and Thermo Fisher Scientific, unless otherwise indicated.

### 2.2. Cell Culture

4T1 (murine triple negative breast cancer cells, CRL-2539), LL/2 (murine lung cancer cells, CRL-1642), CT26.WT (murine colorectal cancer cells, CRL-2638), B16-F10 (murine melanoma cells, CRL-6475) and MDA-MB-231 (human triple negative breast cancer cells, HTB-26) were obtained from the American Type Culture Collection (ATCC, Manassas, VA, USA). HaCaT (non-malignant human keratinocytes, 300493 [53]), BT-474 (human luminal B type breast cancer cells [ER+; PR+; Her-2+], 300131), T-47D (human luminal A type breast cancer cells [ER+; PR+; Her-2−], 300353), SK-BR-3 (human breast cancer cells; HER-2 positive, 300333), A549 (human non-small cell lung cancer cells, 300114), Calu-1 (human squamous lung cancer cells, 300141), MeWo (human melanoma cells; BRAF WT, 300285), NIH:OVCAR-3 (human ovarian cancer cells, 300307) and SK-OV-3 (human ovarian cancer cells, 330342) were purchased from the Cell Line Service (CLS, Hamburg, Germany). UACC-62 (human melanoma cells; BRAF mut) was obtained from the National Cancer Institute (Rockville, MD, USA). HT29 (human colorectal cancer cells) were generously provided by Dr. Helleday (Karolinska Institute, Sweden). ID8 *Trp53*^−/−^ (murine ovarian cancer cells) were a gift from Dr. Iain A. McNeish (Institute of Cancer Sciences, University of Glasgow, UK [54]). 4T1, CT26.WT, T-47D, Calu-1, NIH:OVCAR-3 and UACC-62 were cultured in RPMI 1640. LL/2, B16-F10, MDA-MB-231, HaCaT, BT-474, SK-BR-3, A549, MeWo, SK-OV-3, HT29 and ID8 *Trp53*^−/−^ were grown in Dulbecco’s modified Eagle’s medium (DMEM) high glucose medium. All media were supplemented with 100 U/mL penicillin, 100 μg/mL streptomycin and 10% fetal bovine serum (FBS), except medium for ID8 *Trp53*^−/−^, which was supplemented with 0.11 g/L sodium pyruvate, 4% FBS and 1% insulin–transferrin–selenium. All cells were cultured in a humidified 37 °C incubator with 5% CO_2_.

### 2.3. In Vitro Experiments

Exponentially growing cells were seeded in 96-well plates. After 24 h, the medium was removed and replaced by amino acid-manipulated media (see details below), by complete medium (controls), or by complete medium with several concentrations of an anticancer drug. The cells were visualized daily under a microscope and photographed (20× magnification) on the third and seventh days of treatment using a Huawei P9 lite Leica camera adapted to an inverted microscope. After the treatment period, cell viability was estimated with the MTT assay or the resazurin assay. The MTT is a colorimetric assay based on the capacity of viable cells to reduce yellow tetrazolium MTT (3-(4,5-dimethylthiazol-2-yl)-2,5-diphenyltetrazolium bromide) into an insoluble and purple-colored formazan product. At 24 h after seeding, the cells were exposed to several concentrations of anticancer drugs or to artificial medium. After the treatment period, the medium was removed and 125 μL of MTT diluted in medium (1 mg/mL) was added to the wells. The plates were incubated for 2–4 h at 37 °C, 5% CO_2_. Then, 80 μL 20% SDS in 0.02 M HCl were added to the plates, which were incubated overnight at 37 °C. Finally, optical densities were measured at 540 nm on a multiwell plate spectrophotometer reader. The resazurin assay is a redox-based fluorometric/colorimetric technique based on the capability of viable cells to reduce blue reagent resazurin into the pink, fluorescent and soluble product resorufin. At 24 h after seeding, the cells were exposed to artificial medium for 7 days. The cells were then allowed to recover in their corresponding standard media for 3 days. After treatments and recovery period, medium was removed and 150 μL of resazurin solution (20 μg/mL in medium) was added to each well for 5–7 h (depending on the cell line). The optical densities of each well were measured at 540 nm and 620 nm on a multiwell plate spectrophotometer reader. In both assays, the results were expressed as percentages of cell viability in relation to untreated cells grown in their standard medium. Data for the artificial media were averaged from at least three independent experiments. The data for the anticancer drugs were averaged from two independent experiments and were expressed as the means ± standard error of the mean (SEM).

Artificial media lacking AAs were prepared as described previously [51,52]. Briefly, powdered DMEM medium without AAs (D9800-13; US Biological, Salem, MA, USA) was supplemented with sodium bicarbonate (Panreac, 141638.1211), glucose (Panreac, 141341.1211), FBS, penicillin/streptomycin and specific AAs (see Table 1). 

### 2.4. Animals

Female BALB/cAnNRj mice and female C57BL/6JRj mice (10 weeks or older) were purchased from Janvier Labs^®^ (Le Genest-Saint-Isle, France). To allow adequate acclimation, they were housed in our animal laboratory facilities for at least two weeks before starting the experiments. The animals were kept in standard conditions (12 h light/12 h dark cycle, 70–75% humidity, 24 °C, with ad libitum access to food and water). The mice were fed a standard diet (ssniff diet R/M-Z E/R/S; V1724-000, ssniff Spezialdiäten, Soest, Germany). All mice were 12 weeks or older at the beginning of the experiments.

The experiments were approved by the Animal Ethics Committee of the University of Seville (CEEA-US2018-6/2 and CEEA-US2019-20) and Junta de Andalucía (15/05/2018/090 and 13/11/2020/131). They were carried out under the recommendations of the European Union on animal experimentation (Directive of the European Counsel 2010/630/EU).

### 2.5. In Vivo Cancer Models

In all cancer models, murine cells (5th–7th passage) were cultured in 75-cm^2^ flask until approximately 60–70% confluence. Medium was removed and cells were washed twice with sterile PBS. The cells were then incubated with trypsin/EDTA solution for 1–3 min at 37 °C to allow the cells to have a rounded shape but without detaching. Next, the trypsin/EDTA solution was aspirated, cells were resuspended in 5 mL of sterile PBS and the cell suspension was pipetted up and down to break up any cell aggregate before adding 2.5% FBS supplemented medium. Then, a working cell suspension (between 5 × 10^5^–25 × 10^6^ cells/mL depending on the cancer model) was prepared. The cell suspension was centrifuged (250 g) at room temperature for 5 min. The medium was then removed, and cells were resuspended in warm sterile filtered PBS. Cells were counted again to ensure that the cell suspension was at the correct density. Finally, a 1 mL syringe (insulin type with a 29-G × 1/2″ needle) was filled with 0.2 mL of the working cell suspension, which was injected into the tail vein or into the peritoneal cavity of the mice. One day before initiating the treatments, mice were housed in individual cages to avoid cannibalism. Treatments started four, eight or twenty-one days (depending on the model) after injecting the cancer cells so that they had time to adapt to the new environment and proliferate before the beginning of the treatments. The aim of our research was not to prevent metastasis, but to find new treatments for patients with stablished metastatic cancers. Untreated animals (control group) continued to be fed with their standard diet (ssniff diet). Other groups of mice received drugs used in patients with the selected type of cancer. In the groups of mice treated with the artificial diets, treatment consisted of replacing their normal diet with one of the artificial diets. Treatments with the artificial diets lasted at least 4 weeks, unless otherwise specified or shown. The in vivo cancer models used in this work [54,55,56,57,58,59,60,61] are summarized in Table S1.

In the triple negative breast cancer model, female BALB/cAnNRj mice were inoculated with 10^5^ 4T1 cancer cells in the tail vein [55,56]. Treatments started 8 days after the injection of the cells. Doxorubicin and capecitabine were used as positive controls. Doxorubicin (0.5 mg/kg) was injected intraperitoneally once a week for 4 weeks. Capecitabine (450 mg/kg/day) was administered in the diet following a 7/7 on/off schedule; the animals received 2 or 3 cycles depending on their state of health. To follow the recommendations of the Animal Ethics Committee, treatments were initially screened using 2–4 mice per group. The anticancer activity of the active treatments was then evaluated in independent experiments with a higher number of animals. To facilitate comparison between groups, mice receiving the same treatment were merged in the survival curves. Therefore, survival data of mice included in the control or positive control groups in the screening experiments were used for the preparation of the survival curves of several active treatments. All experiments included a control group (untreated mice) and a positive control group (doxorubicin or capecitabine).

The colon cancer model was established by injecting 10^5^ CT26.WT cells in the peritoneal cavity (peritoneal dissemination model) or in the tail vein (lung metastasis model) of female BALB/cAnNRj mice [57,58]. In both models, treatments started 4 days after cancer cell inoculation. Capecitabine (450 mg/kg/day) was administered in the diet following a 7/7 on/off schedule; the animals received 2 or 3 cycles depending on their state of health.

In the lung cancer model, 2 × 10^6^ LL/2 cells were injected in the tail vein of female C57BL/6JRj mice [59,60]. Treatments started 7 days after the inoculation of the cancer cells. Anti-PD-1 was used as a positive control; it was administered intraperitoneally every 4 days for a total of 4 doses. In each dose, mice received 250 μg anti-PD-1 diluted in pH 7.0 buffer (InVivoPure, IP0070, Bioxcell).

The ovarian cancer model was established by injecting 5 × 10^6^ ID8 *Trp53*^−/−^ cells into the peritoneal cavity of female C57BL/6JRj mice [54]. Treatments started 21 days after the inoculation of the cancer cells. The positive control cisplatin (5 mg/kg) was administered intraperitoneally once a week for 4 weeks.

The melanoma cancer model was established by inoculating 10^6^ murine B16-F10 melanoma cells in the tail vein of female C57BL/6JRj mice [61]. Treatments started 4 days after the injection of the cancer cells. Cisplatin was used as a positive control. Cisplatin (5 mg/kg) was administered intraperitoneally once a week for 4 weeks.

All animals were monitored daily, and body weights were recorded periodically (at least three times per week). Mice were sacrificed by cervical dislocation when signs of cancer progression were apparent; these signs (e.g., respiratory distress and reduced curiosity and mobility) indicated that survival for an additional 2 days was unlikely (Table S2). Necropsy was performed to verify the cause of death and to observe the extent of the disease. The presence of tumors was confirmed in all sacrificed mice, and similar tumor loads were observed unless otherwise specified or shown. Lungs were dyed with India ink and fixed with Fekete’s solution (100 mL of 70% ethanol, 5 mL of 100% glacial acetic acid and 10 mL of 4% formaldehyde). With this technique, tumors show a white appearance and normal lung parenchyma appears black. In the melanoma model, lungs were not dyed with India ink because melanoma tumors have a natural black appearance.

### 2.6. Artificial Diet Preparation and Composition

All artificial diets (Table 2) were prepared in our laboratory. All solid ingredients were mixed until they formed a well-blended dry powder. The oil was then added to the mixture, and enough water was slowly added until a soft dough was formed. The dough was air dried for 2 h, manually pelleted, air-dried for an additional 24 h, and stored at room temperature until use. Fresh diets were prepared for each independent experiment.

Casein (bovine casein 27607, Acros Organics) constituted 6% of the dry diets TB4 and TB5. The typical amount (g) of AAs in 100 g and 6 g (shown in brackets) of the casein used in the experiments is Gln + Glu: 21.7 (1.302), Leu: 9 (0.54), Met: 2.9 (0.174), Phe: 4.8 (0.288), His: 2.6 (0.156), Lys: 7.5 (0.45), Thr: 4.1 (0.246), Ile: 4.3 (0.258), Val: 5.3 (0.318), Trp: 1.2 (0.072), Cys/CySS: 0.7 (0.042), Arg: 3.4 (0.204), Gly: 1.7 (0.102), Ser: 5.7 (0.342), Tyr: 5.2 (0.312), Ala: 2.9 (0.174), Asp + Asn: 6.9 (0.414), Pro: 10.1 (0.606).

The dry diets contained 1% Vitamin Mix (AIN Vitamin Mixture 76, MP Biomedical). A total of 100 g of the dry diets contained (mg) thiamine hydrochloride (0.6), riboflavin (0.6), pyridoxine hydrochloride (0.7), nicotinic acid (3), d-calcium pantothenate (1.6), folic acid (0.2), d-biotin (0.02), cyanocobalamin (0.001), retinyl palmitate premix (250,000 IU/g) (1.6), DL-a-tocopherol acetate (250 IU/g) (20), cholecalciferol (400,000 IU/g) (0.25), menaquinone (0.005), sucrose (972.9). The dry diets contained 3.5% Mineral Mix (AIN-93M-MX, MP Biomedical). A total of 100 g of the dry diets contained 1.25% calcium carbonate, 0.875% monopotassium phosphate, 0.098% potassium citrate, 0.259% sodium chloride, 0.163% potassium sulfate, 0.085% magnesium oxide, 0.021% ferric citrate, 0.0058% zinc carbonate, 0.0022% manganese carbonate, 0.0011% copper carbonate, 0.000035% potassium iodate, 0.000035% sodium selenate, 0.000028% ammonium paramolybdate-tetrahydrate, 0.0051% sodium metasilicate-nonahydrate, 0.00095 chromium potassium sulfate-dodecahydrate, 0.0000595% lithium chloride, 0.000284% boric acid, 0.00022% sodium fluoride, 0.00011% nickel carbonate hydroxide, 0.000021% ammonium meta-vanadate and 0.73% sucrose. Tert-butylhydroquinone (150822500, Acros Organics) was used as an antioxidant for the TB2-TB5 diets, comprising 0.0008% of the dry diet.

The Ssniff diet was used as a control diet (SM R/M-S E, 10 mm; V1724-000). This diet contains 21% protein, 7% fat, 4% fiber, 6.2% ash, 33.3% starch and 4.6% sugar.

### 2.7. Statistical Analysis

Results were expressed as mean ± standard error mean (SEM). Statistical analysis was performed with the GraphPad Prism version 7.0 software. Statistical analysis for the Kaplan–Meier survival curve was calculated using the Gehan–Breslow–Wilcoxon (GBW) test. A *p* value > 0.05 is not considered statistically significant and is not represented by any symbol. The *p*-value < 0.05 is considered statistically significant and is indicated with an asterisk (*), <0.01 with two asterisks (**) and <0.001 with three asterisks (***).

## 3. Results

### 3.1. Amino Acid Restriction Induces Selective Anticancer Activity in Breast Cancer Cells In Vitro

Patients with metastatic TNBC need selective anticancer treatments, that is, treatments that can eliminate their cancer cells without significantly affecting their normal cells. We therefore evaluated our anticancer strategy in human TNBC cells (MDA-MB-231) versus human non-malignant cells (HaCaT). We also used murine TNBC cells (4T1) to detect potential experimental artifacts caused by species differences in sensitivity to treatments [62]. We selected a medium lacking 10 NEAAs (all NEAAs except Gln, M1), which previously showed selective cytotoxic activity against colon cancer cells and renal cancer cells [51,52]. This medium lacked all NEAAs except Gln, to force cancer cells to biosynthesize them. Gln was used as a source of amino groups for the biosynthesis of the other NEAAs. Since the anticancer activity of a restriction therapy lacking several components cannot be tested following the standard dose–response approach (e.g., IC50 values cannot be calculated), we used the following experimental approach [51,52]. The three cell lines were grown in the AA-deficient medium (M1) or in a complete medium (M0) for seven days. Cell morphology and density were visualized daily under a microscope, and representative photographs were taken on days 3 and 7. Cell viability was determined on day 7 with the MTT assay. Because doxorubicin and capecitabine, a prodrug of 5-fluorouracil (5-FU), are standard treatments for TNBC patients, we also tested doxorubicin and 5-FU in the same cell lines.

**Figure 1:**
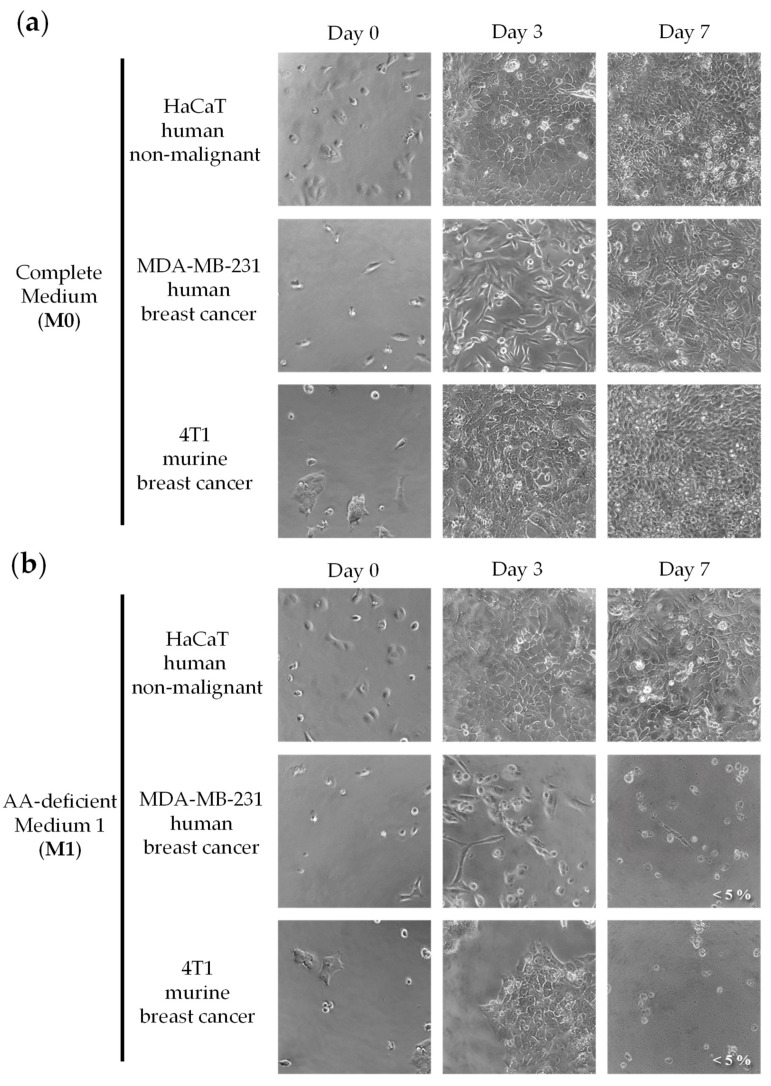
An artificial medium lacking 10 NEAAs induces selective cytotoxic activity in triple-negative breast cancer cells. Cells were grown in a complete medium (M0) or in a medium lacking 10 AAs (M1) for seven days. Cells were monitored by microscopic visualization and photographed on days 3 and 7. Representative photographs at 20× magnification are shown. Cell viability was estimated with the MTT assay and is shown at the bottom right of the photographs when it was less than 10%. The detailed compositions of M0 (**a**) and M1 (**b**) are shown in Table 1.

**Figure 2:**
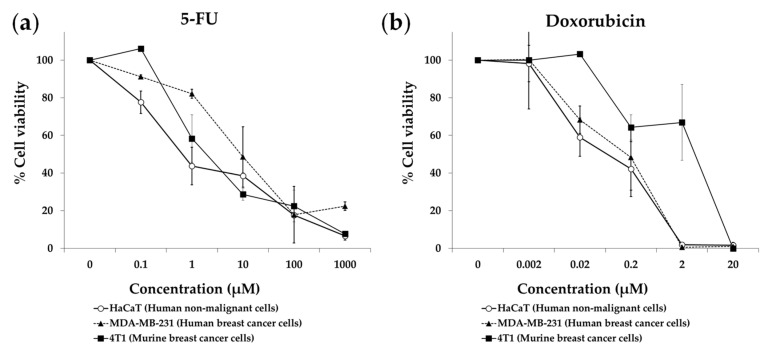
Cytotoxic effect of 5-fluorouracil (**a**) and doxorubicin (**b**) on triple-negative breast cancer cells and non-malignant cells. Cells were treated for 72 h, and cell viability was estimated with the MTT assay. Data show the mean ± SEM of at least 2 independent experiments.

Figure 1 shows that our artificial medium lacking 10 NEAAs (M1) induced selective cytotoxicity against TNBC cells, especially after 7 days of treatment. Non-malignant cells (HaCaT) incubated with M1 proliferated slower than those incubated with M0, but they eventually saturated the wells after 7 days of exposure. Clear antiproliferative effects were observed in both human and murine TNBC cells (MDA-MB-231 and 4T1) incubated for 7 days with M1. Importantly, the standard anticancer drugs doxorubicin and 5-FU did not show selective cytotoxicity in this panel of cell lines. After three days of treatment, both drugs induced potent cytotoxic effects against the cancer cells, but also against the non-malignant cells (Figure 2). The IC50 values (mean ± SEM, µM) for 5-FU were 1.11 ± 0.74 in HaCaT cells, 16.71 ± 12.09 in MDA-MB-231 cells and 1.86 ± 1.03 in 4T1 cells. The IC50 values (mean ± SEM, µM) for doxorubicin were 0.14 ± 0.12 in HaCaT cells, 0.20 ± 0.15 in MDA-MB-231 cells and 3.16 ± 2.17 in 4T1 cells.

Our medium lacking 10 NEAAs (M1) also induced selective cytotoxicity against other types of human breast cancer cells: BT-474 (luminal B type [ER+; PR+; Her-2+]), T-47D (luminal A type [ER+; PR+; Her-2-]) and SK-BR-3 (HER-2 positive) (Table S3 and Figure S1). These data indicate that the selective anticancer activity of our restriction therapy is not limited to TNBC cells.

### 3.2. Anticancer Activity of an Artificial Diet Lacking 10 NEAAs in Mice with Metastatic Triple-Negative Breast Cancer

**Figure 3:**
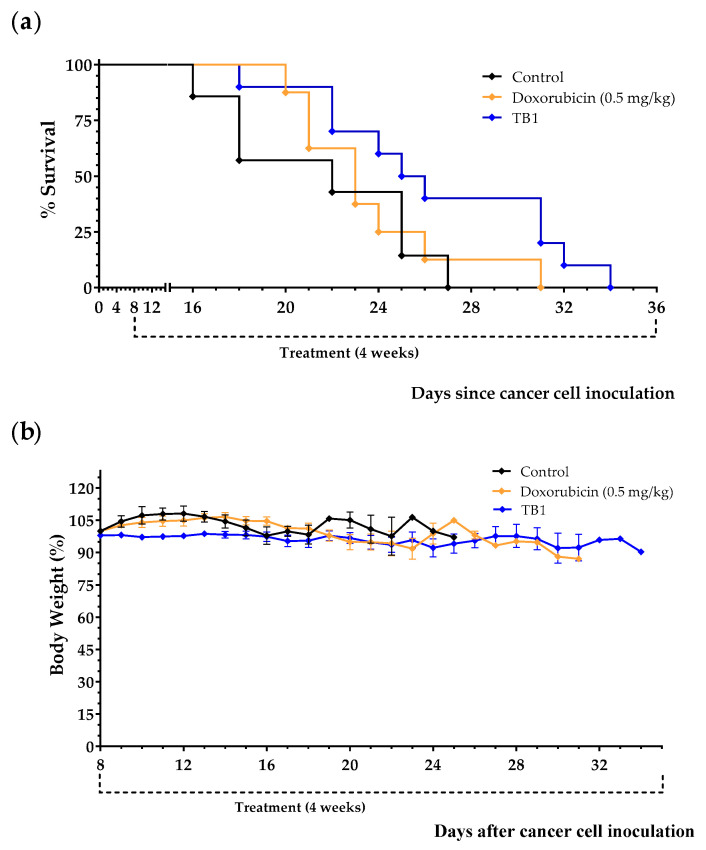
Anticancer effect of diet TB1 and doxorubicin in mice with TNBC (4T1 murine breast cancer cells inoculated in the tail vein of immunocompetent BALB/c mice). Survival (**a**) and body weight (**b**) of mice treated with doxorubicin (0.5 mg/kg/day, every week for a total of 4 doses), with diet TB1 (normal diet was replaced by TB1 for 28 days) or untreated mice (control, normal diet). Treatments began 8 days after the intravenous injection of 100,000 4T1 cancer cells. Body weight are expressed as percentage relative to the body weight at the beginning of the treatments (day 8). Data are from three independent experiments. The four mice fed diet TB1 that lived longer are from two independent experiments.

**Figure 4:**
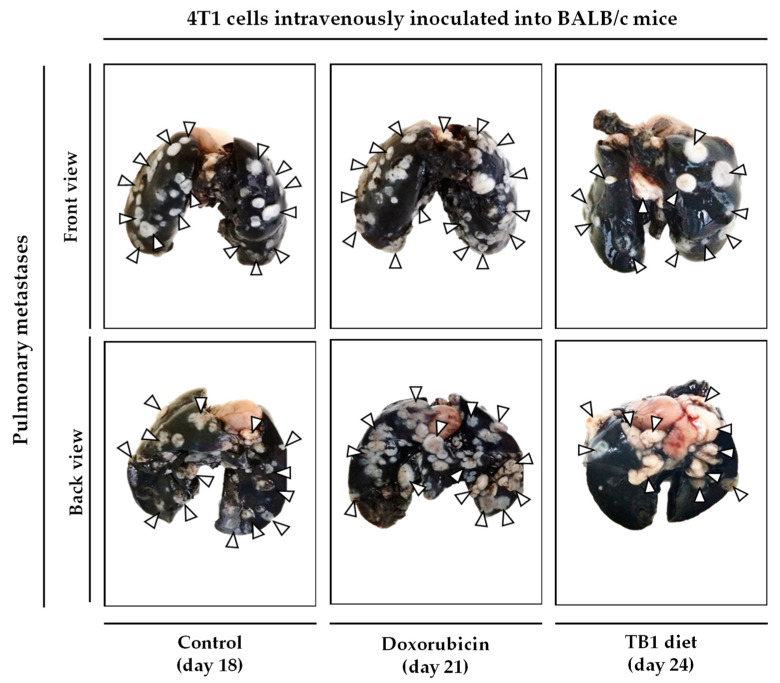
Lung photographs at the time of sacrifice of representative mice with TNBC. In this model, 100,000 4T1 murine breast cancer cells are inoculated in the tail vein of immunocompetent BALB/c mice. After 8 days, mice were treated with doxorubicin (0.5 mg/kg/day, every week for a total of 4 doses), with diet TB1 (normal diet was replaced by TB1 for 28 days) or untreated (control, normal diet). Mice were euthanized by cervical dislocation when signs of disease progression were apparent. After sacrifice, lungs were excised and stained with India ink (tumors show a white appearance and normal lung parenchyma appears black; some photographs show the unstained heart). Mice were sacrificed at different time points, when symptoms of advanced disease were patent. The day of sacrifice is shown in brackets.

Since the medium lacking 10 NEAAs (M1) induced selective anticancer activity in TNBC cells in vitro, we prepared an artificial diet lacking the same AAs (diet TB1) to test its anticancer activity in an in vivo model of metastatic TNBC. The model was established by injecting murine TNBC cells (4T1) into the tail vein of immunocompetent female BALB/cAnNRj mice. In this model, all untreated mice die several weeks after the inoculation of the cancer cells. Treatments began 8 days after the injection of the cancer cells. Mice were euthanized by cervical dislocation when signs of disease progression were apparent; these signs (e.g., respiratory distress and/or reduced mobility and curiosity) indicated that survival for an additional 48 h was unlikely. The postmortem examination confirmed the presence of tumors in all euthanized mice, mainly in the lungs. Doxorubicin, a standard treatment for patients with metastatic breast cancer, was used as a positive control. Table 3 and Figure 3 show the results from three independent experiments. Diet TB1 was well tolerated and modestly increased mice survival; mean survival of mice fed diet TB1 was approximately 5 days longer than control mice (Figure 3). Most mice fed our artificial diet lived longer than mice treated with doxorubicin. This is a challenging in vivo model in which standard therapies induce a low activity. Figure 4 shows lung photographs at the time of sacrifice of representative mice from each group. Untreated mice and mice treated with doxorubicin had a similar number of tumors with similar size. Mice fed diet TB1 generally had fewer tumors, but some of them were bigger. Possibly, diet TB1 inhibited the proliferation of some tumors, but others continued to grow until they caused respiratory distress in the animals.

### 3.3. An Artificial Diet without 10 NEAAs and with 1% Lipids Induces Anticancer Activity in Mice with Metastatic Triple-Negative Breast Cancer

We have recently reported that several artificial diets with low levels of lipids induced marked anticancer activity in mice with renal cell carcinoma and colon cancer. [51,52]. To evaluate the impact of reducing lipid levels on TNBC progression, mice inoculated with 4T1 cells were treated with diet TB2; this diet was prepared by reducing the lipid levels of diet TB1 from 14% to 1% (Table 2). Treatments began 8 days after the intravenous inoculation of the 4T1 cancer cells. Animals were treated with oral capecitabine (450 mg/kg/day), with diet TB2 (the normal diet was replaced by this diet for 28 days) or were left untreated (control group).

**Figure 5:**
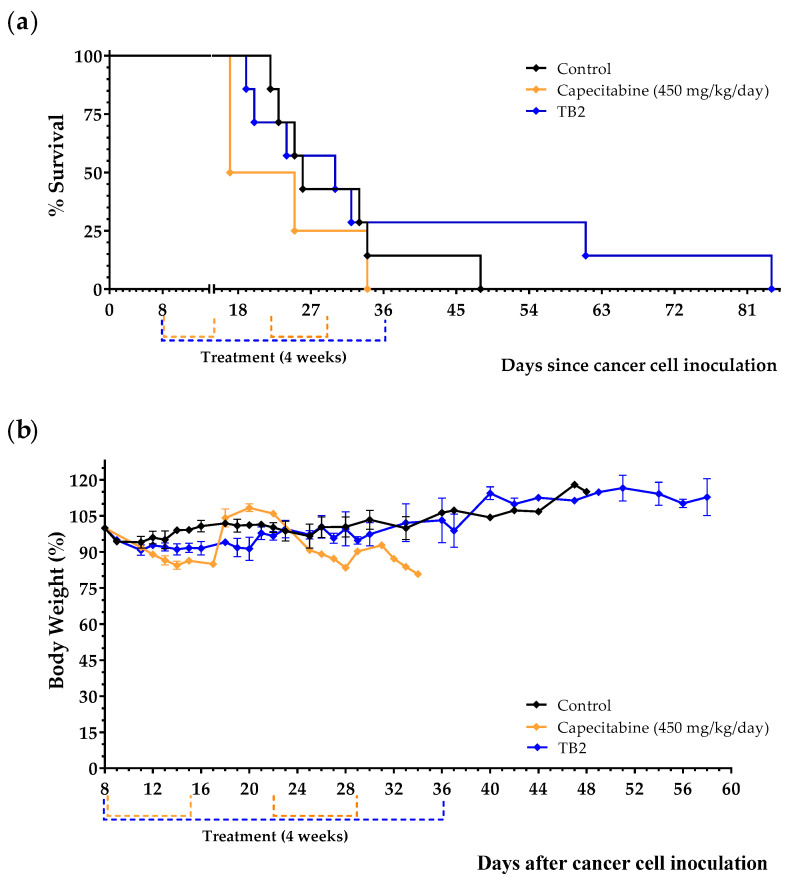
Anticancer effect of diet TB2 and capecitabine in mice with metastatic TNBC. Survival (**a**) and body weight (**b**) of mice treated with diet TB2 (normal diet was replaced by TB2 for 28 days) or with capecitabine (450 mg/kg/day, 7/7 schedule, 3 cycles). Treatments started 8 days after cancer cell inoculation. Body weight are expressed as percentage relative to the body weight at the beginning of the treatments (day 8). See Table 2 for the composition of the diets and the main text for further details. Data are from two independent experiments. The two mice fed diet TB2 that lived longer are from the same experiment.

Results, shown in Table 4, Figure 5a and Figure S2, indicate that diet TB2 induced a marked anticancer activity in two of the seven mice; these two mice were sacrificed on days 61 and 84. Importantly, diet TB2 was well tolerated, and mice did not suffer significant weight losses despite the drastic reduction in lipid levels. Mice treated with capecitabine showed marked adverse effects (e.g., decreases in body weight and decreases in spontaneous motor activity) that reverted at the end of each treatment cycle (Figure 5b). In these experiments, capecitabine was completely inactive. These results indicate that lipid levels can markedly increase mice survival in a low percentage of animals with metastatic TNBC fed an artificial diet lacking NEAAs.

### 3.4. An Artificial Diet without 10 NEAAs, with Reduced Levels of EAAs, and with 1% Lipids (Diet TB3) Induces Anticancer Activity in Mice with Metastatic Triple-Negative Breast Cancer

Diets TB1 and TB2 lack 10 NEAAs; however, these diets contain high levels of all essential AAs (EAAs). Evidence suggests that restriction of some EAAs (e.g., methionine) induces anticancer activity in TNBC models [24,25,26,27]. We therefore sought to improve the anticancer activity of our artificial diets by reducing their levels of EAAs. In diet TB3, all EAAs except Leu were reduced by a factor of approximately 3.5 with respect to diets TB1 and TB2. Keeping high Leu levels may be important to prevent proteolysis [63,64]. Diet TB3 contained 1% coconut oil instead of 1% olive oil to reduce the levels of monounsaturated fatty acids (MUFA), which may protect cancer cells against ferroptotic cell death [65].

**Figure 6:**
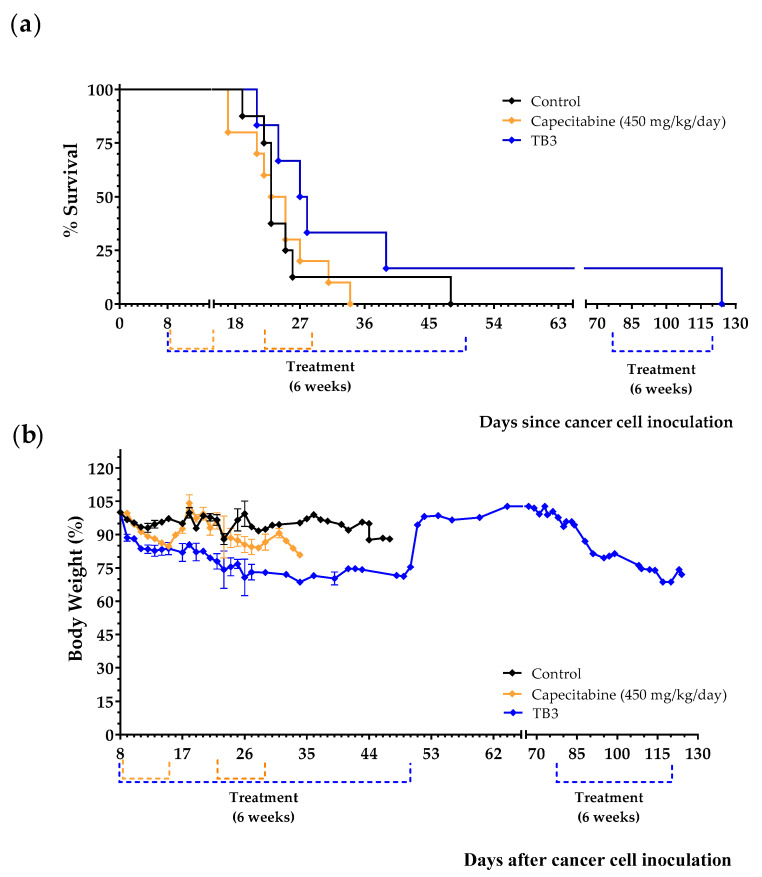
Anticancer activity of diet TB3 and capecitabine in mice with metastatic TNBC. (**a**) Survival of mice treated with diet TB3 (normal diet was replaced with this diet), or oral capecitabine (450 mg/kg/day). (**b**) Body weights (mean percentage ± SEM) relative to body weights at the start of the treatments (day 8). See Table 2 for the composition of the diet and the main text for further details. Data are from two independent experiments.

Treatments began 8 days after the tail vein injection of the 4T1 cancer cells. Animals were treated with diet TB3 (the normal diet was replaced by this diet for 6 weeks), with capecitabine (450 mg/kg/day, 7/7 schedule, 3 cycles) or were left untreated (control group). Mean survivals were 26.1 ± 3.2 in untreated mice, 43.8 ± 16.2 in mice fed diet TB3 and 24.2 ± 1.7 in mice treated with capecitabine. Most mice treated with diet TB3 lived longer than untreated mice (Figure 6a). One of the mice treated with diet TB3 survived the initial 6-week treatment and, after coming back to a normal diet for 4 weeks, treatment was restarted for an additional 6-week period. This animal was sacrificed on day 124 with cancer-related symptoms (decreased spontaneous motor activity and accelerated breathing), and the autopsy confirmed the presence of several tumors in the lungs. Although diet TB3 was well tolerated, body weights decreased continuously during treatment. Capecitabine also induced significant weight losses that also reverted at the end of each treatment cycle (Figure 6b).

### 3.5. Diet TB3 Induces Anticancer Activity in Mice with Other Types of Metastatic Cancers

To test if the marked in vivo anticancer activity of diet TB3 was specific for BALB/cAnNRj mice inoculated with 4T1 cells, and to screen its therapeutic potential for other types of cancer, we tested the anticancer activity of diet TB3 in other metastatic cancer models. We first carried out several in vitro experiments and observed that our artificial medium M1 also induced selective cytotoxicity in lung cancer cells, colorectal cancer cells, ovarian cancer cells, and melanoma cells versus human non-malignant cells (Figure S3). Then, we evaluated diet TB3 in mice with several types of metastatic cancers: lung cancer (intravenous injection of LL/2 cancer cells in C57BL/6JRj mice), colon cancer (intraperitoneal or intravenous injection of CT26WT cancer cells in BALB/cAnNRj mice), ovarian cancer (intraperitoneal injection of ID8 *Trp53*^−/−^ cancer cells in C57BL/6JRj mice) and melanoma (intravenous injection of B16-F10 cancer cells in C57BL/6JRj mice). We used 3–4 mice per group in each cancer model to follow the Animal Ethics Committee recommendations and limit the number of mice to a minimum. A total of 16 mice with different types of metastatic cancers were treated with diet TB3 in these experiments. Treatments started 4 days after the injection of the cancer cells in the two colon cancer models, in the lung cancer model and in the melanoma model. Treatments began 21 days after the injection of the cancer cells in the ovarian cancer model; this model progresses slower than the other cancer models and treatments can be initiated later. We used capecitabine, cisplatin or anti-PD-1 as positive controls.

**Figure 7:**
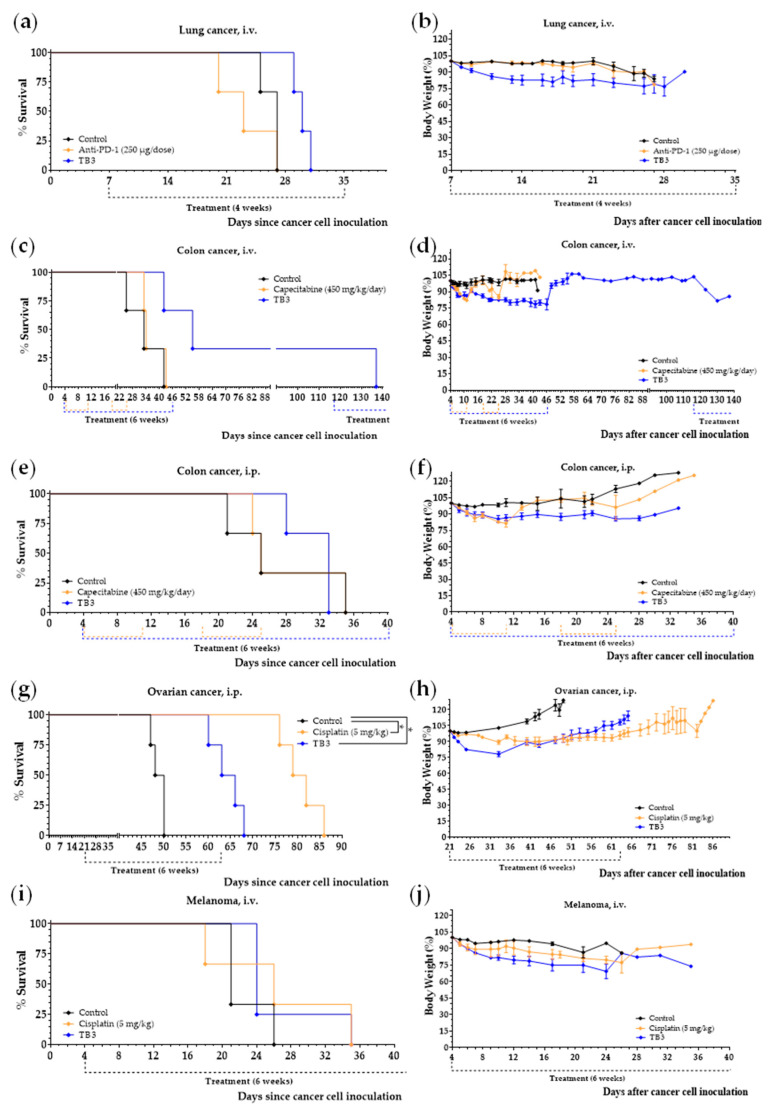
Diet TB3 induces marked anticancer activity in mice with different types of metastatic cancer. Survival of mice left untreated (control), treated with diet TB3 (normal diet was replaced with this diet), or treated with intraperitoneal anti-PD-1 (250 µg/dose), oral capecitabine (450/mg/day) or intraperitoneal cisplatin (5 mg/kg). Survival (**a**,**c**,**e**,**g**,**i**) and body weights (**b**,**d**,**f**,**h**,**j**) of mice with metastatic cancers treated with diet TB3 or a standard anticancer drug. The *p*-value was calculated with the Gehan–Breslow–Wilcoxon test. See text for further details.

Diet TB3 improved mice survival in all the cancer models (Table 5 and Figure 7a,c,e,g,i). In the lung cancer model, melanoma model and intraperitoneal colon cancer model, mice fed diet TB3 lived approximately 4 days longer than untreated mice. Although this survival improvement was moderate, the activity of the standard treatments was similar (melanoma) or worse (lung cancer and intraperitoneal colon cancer). All mice inoculated intravenously with the colon cancer cells and treated with diet TB3 lived longer than untreated mice; the survival improvement was 44.7 days. One of the mice with colon cancer survived the initial 6-week treatment. The mouse developed disease symptoms and diet TB3 was restarted on day 116. Despite receiving diet TB3, the disease finally advanced and the mouse was sacrificed on day 137. In the ovarian cancer model, all mice fed diet TB3 lived longer than untreated mice (mean survival improvements was 15.5 days). However, the positive control cisplatin was much better. Since cisplatin was administered intraperitoneally, it may exert a direct cytotoxic effect on the ovarian cancer cells (which were also inoculated in the peritoneal cavity); this may contribute to explaining the high activity of cisplatin in this cancer model. 

**Figure 8:**
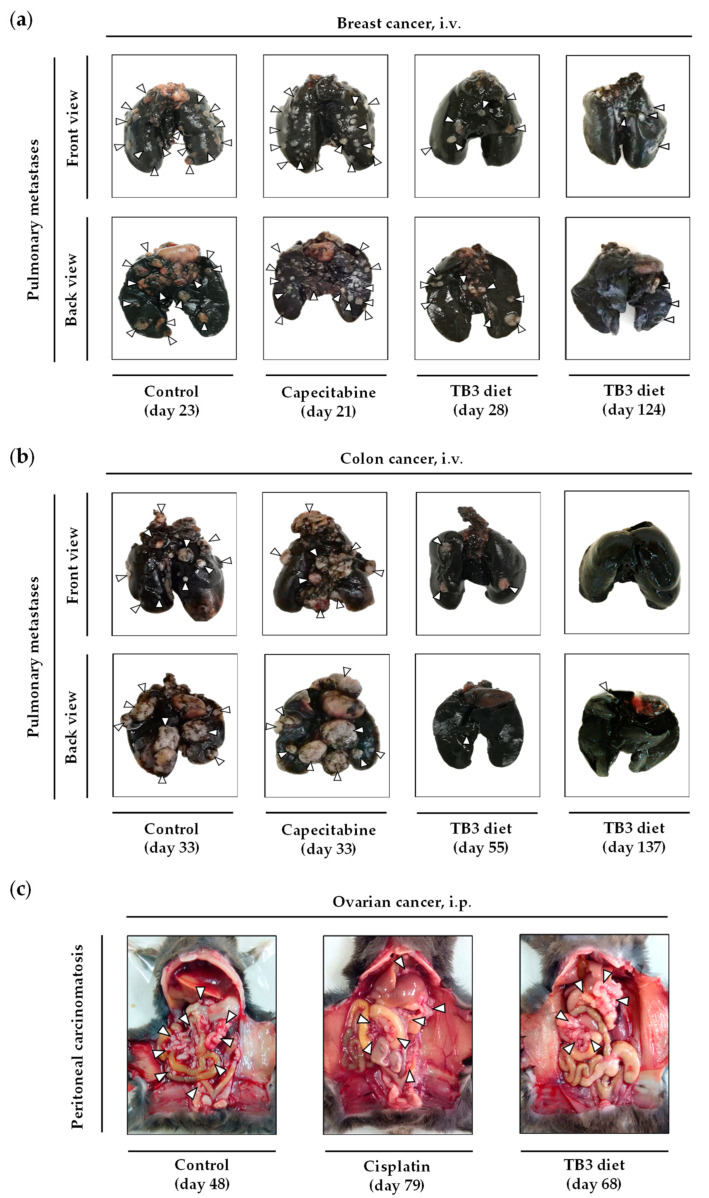
Representative photographs at the time of sacrifice of mice with different types of metastatic cancers. In these models, mice were treated with diet TB3 (normal diet was replaced by TB3 for 6 weeks), with a standard anticancer drug or did not receive any treatment (control, normal diet). In the models of TNBC (**a**) and colon cancer (**b**), the lungs were excised and stained with India ink (tumors show a white appearance and normal lung parenchyma appears black). In the ovarian cancer model, representative photographs of the peritoneal cavity are shown (**c**). The day of sacrifice is shown in brackets.

A total of 23 mice with different types of metastatic cancers (including TNBC) were treated with diet TB3 (normal diet was replaced by this artificial diet). The total mean survival was 30.5 ± 2.2 days for untreated mice and 45.4 ± 6.4 days for mice treated with diet TB3 (Table 5). Figure 8 and Figure S4 show representative photographs of mice in all these cancer models; the aggressiveness of these models may explain why none of the animals were cured by any standard or experimental treatment. In all these models, the inoculation of the cancer cells led to the development of multiple tumors in the animals. In the lung metastasis models (Figure 8a,b and Figure S4a,b), necropsies showed numerous tumors in the lungs of most mice. The number of tumors was generally higher in animals with short survival times. Mice with longer survival times generally showed fewer but bigger tumors, which eventually compromised respiratory function. In the lung metastasis models, tumors outside the thoracic cavity were also observed in some mice with long survivals. In the peritoneal dissemination models (Figure 8c and Figure S4c), necropsies showed that animals with longer survival times generally had fewer but bigger tumors in the peritoneal cavity. Diet TB3 reduced mice body weight in all cancer models (Figure 7b,d,f,h,j).

### 3.6. Diets TB4 and TB5 (Artificial Diets with 6% Casein and 1% Lipids) Induce Anticancer Activity in Mice with Metastatic Triple-Negative Breast Cancer

**Figure 9:**
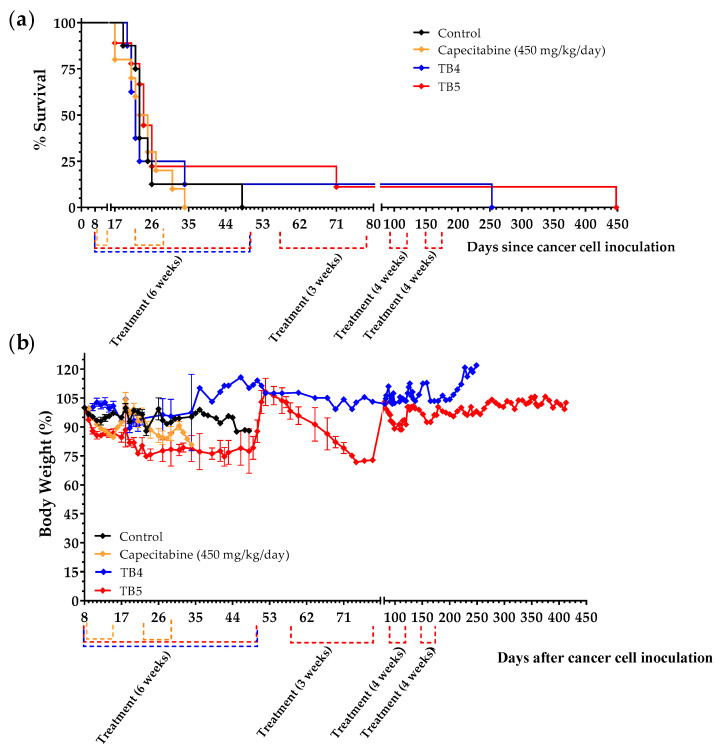
Anticancer activity of diets TB4 and TB5 and capecitabine in mice with metastatic TNBC cancer (4T1 murine breast cancer cells inoculated in the tail vein of immunocompetent BALB/c mice). (**a**) Survival of mice left untreated (control), treated with diet TB4 (6 weeks), treated with diet TB5 (6 weeks) or treated with capecitabine (450 mg/kg/day, 7/7 schedule, 3 cycles). (**b**) Body weights (mean percentage ± SEM) relative to the body weights of mice at the beginning of treatments (day 8). The two mice fed diet TB5 that lived longer are from the same experiment.

We next prepared diets TB4 and TB5 to continue exploring the role of manipulating AAs and lipids on the progression of mice with metastatic TNBC. These two diets contain low levels of the protein casein (6%), low lipid levels (1% coconut oil in diet TB4 and 1% salmon oil in diet TB5) and a 5% Gln supplement. Diet TB5 also contains a 5% Leu supplement (Table 2). We chose casein because this protein provides low levels of the sulfur-containing AAs Cys and Met. We have previously observed that controlling the levels of Cys, Met, Gln and Leu could change the activity of the artificial diets in mice with renal and colon cancers [51,52]. Because the balance between saturated and unsaturated fatty acids may play a role in the survival of cancer cells [66], we used coconut oil (rich in saturated fatty acids) or salmon oil (rich in unsaturated fatty acids). Treatments began 8 days after the inoculation of 4T1 cancer cells into the tail vein of immunocompetent BALB/c mice. Animals were left untreated (control group) or were treated with capecitabine (450 mg/kg/day), diet TB4 or diet TB5 (the normal diet was replaced with one of these diets for 6 weeks). Both diets induced anticancer activity in mice with metastatic TNBC (Figure 9a and Table 6). One mouse treated with diet TB4 had a very long survival; it was sacrificed on day 253 with cancer-related symptoms, and the autopsy revealed the presence of a metastatic tumor in the peritoneal cavity and a marked splenomegaly (Figure S5). This diet was well tolerated, and mice did not suffer significant weight loss (Figure 9b). Two mice fed diet TB5 survived the initial treatment and received additional treatment cycles (normal diet was replaced with TB5 diet) when cancer-related symptoms appeared. One of the mice developed advanced disease symptoms and was sacrificed on day 71; the autopsy confirmed the presence of a metastatic tumor in the lungs and several small tumors in the thorax. The other mouse was treated several times with TB5 and was eventually sacrificed on day 448 because of the appearance of persistent blood in the urine. The autopsy confirmed the presence of two metastatic tumors in the peritoneal cavity (Figure S5). Diet TB5 induced a marked weight loss that reverted after treatment. Several cycles of the standard anticancer drug capecitabine did not improve the survival of mice with metastatic TNBC. Our previous data revealed that diet TB5 (also denominated diet TC7) was ineffective in several mice with metastatic colon cancer [51].

## 4. Discussion

Most patients diagnosed with metastatic TNBC do not overcome the disease. The available pharmacological treatments can prolong patient survival and palliate disease-related symptoms, but they are rarely curative. The aim of this work was to evaluate the anticancer potential of artificial diets based on selective restriction of AAs in mice with metastatic TNBC.

In addition to acquiring DNA alterations [67], TNBC cells develop metabolic changes that may be exploited therapeutically [8,9,10]. Other research groups have previously shown that limiting the levels of specific AAs with AA-depleting enzymes or through dietary restriction induced in vivo anticancer effects in TNBC models [19,20,24,25,26,27,28,29]. However, none of these studies have shown major improvements in the survival of animals with metastatic TNBC. In this work, we took a different approach to exploit the altered metabolism of cancer cells and increase the efficacy of this therapeutic strategy. Instead of restricting the levels of a particular AA, we created massive changes in AA levels and ratios to generate challenging metabolic environments for cancer cells. As discussed previously [64], cancer cells have mutations and other DNA changes that provide them with a survival advantage under a standard physiological environment. However, these same DNA alterations may cause their death in a different environment, because the survival of cancer cells depends not only on the acquisition of beneficial DNA changes, but also on favorable environments for these DNA changes. Since all cancer cells have originated under environments in which the levels and ratios of the 20 proteinogenic AAs are relatively constant, changing these levels and ratios with artificial diets may create new and unfavorable metabolic environments for cancer cells. Under these new metabolic environments, the DNA aberrations of cancer cells may become a liability that leads to their selective death. Normal cells have a functional DNA and may therefore resist the temporal AA imbalances created with artificial diets [64].

To test the therapeutic potential of our anticancer strategy, we used an experimental approach focused on cancer patients’ needs [68,69]. Cancer patients need treatments that can eliminate their cancer cells without significantly affecting their normal cells. The existing anticancer drugs can kill cancer cells through a variety of mechanisms of action; however, they also kill normal cells at similar concentrations. This implies that cancer patients cannot receive the drug doses required to eliminate their cancer cells, because these doses would also eliminate their normal cells and would be lethal. Patients receive tolerable doses rather than effective doses, which are insufficient to cure the disease in most cases. To be therapeutically useful, an experimental treatment must be selective towards cancer cells, and its selectivity should be higher than that of the existing therapies. We therefore initiated our investigation by evaluating if our experimental treatment could kill TNBC cells without significantly affecting non-malignant cells. Then, we evaluated if its selectivity was higher than that of drugs used in patients with TNBC. We prepared an artificial medium lacking 10 AAs (M1 medium) and observed that its selective anticancer activity (Figure 1) was higher than that of doxorubicin and 5-FU (Figure 2). These two drugs actually lacked selectivity toward cancer cells, probably because the non-malignant cells used in the experiments have high proliferative rates, and chemotherapy drugs also target normal cells with high proliferative rates. M1 medium also induced selective anticancer activity against other types of human breast cancer cells (Figure S1).

Our in vitro experiments proved that TNBCs and other types of breast cancer cells can be selectively killed by manipulating AA levels. However, these results should be interpreted cautiously, because the metabolic environment of cells growing in vitro and in vivo is extremely different. For example, in our artificial medium, the concentration of 10 of the 20 proteinogenic AAs before adding FBS was 0%. These low concentrations cannot be achieved in the systemic circulation of patients, because the liver and muscles can provide AAs to ensure that their plasma levels are not so drastically reduced [64]. We therefore continued our investigation by using in vivo experiments.

Patients with metastatic TNBC need curative treatments or, at least, treatments that improve the survival rates achieved with the existing therapies. We therefore selected an animal model of metastatic TNBC to evaluate if our artificial diets were curative or, at least, better than the standard treatments [68]. We prepared a diet lacking the same 10 NEAAs as medium M1 (diet TB1) and treated the animals by replacing their normal diet with this artificial diet. Several independent experiments showed that mice with metastatic TNBC fed diet TB1 lived several days longer than untreated mice and mice treated with doxorubicin (Figure 3). However, the survival improvements achieved with diet TB1 were mild. The activity of this diet (also known as diet T1) was also mild in mice with renal cell carcinoma [52]. We then screened several diets with other AA combination (2–3 mice per group), without observing improvements in the survival of mice with TNBC (results not shown). However, when we reduced the lipid levels of diet TB1 from 14% to 1% to create diet TB2, a marked survival improvement was observed in two mice (they lived several weeks longer than untreated mice; Figure 5).

Diets TB1 and TB2 lacked 10 NEAAs but contained high levels of all EAAs. Since EAAs can facilitate tumor progression [70], we sought to improve the anticancer activity of our diets by reducing the levels of EAAs. In diet TB3, the levels of all EAAs except Leu were reduced, and lipid levels were kept at 1% (Table 2). One mouse treated with diet TB3 had a very long survival; it was sacrificed 124 days after the inoculation of the cancer cells (Figure 6). To exclude the possibility that this high activity could have been artificially increased by our experimental model (4T1 cells inoculated in the tail vein of BALB/c mice), and to evaluate the therapeutic potential of this diet in mice with other types of cancer, we screened diet TB3 in several animal models of metastasis. The results revealed that diet TB3 prolonged mice survival in all the selected cancer models. The activity was moderate in the lung cancer model, melanoma model and intraperitoneal colon cancer model. In the intravenous colon cancer model, one mouse lived 137 days after the inoculation of the cancer cells. In the intraperitoneal ovarian cancer model, all four C57BL/6 mice fed diet TB3 lived approximately 2 weeks longer than untreated mice. In this model, treatments started 21 days after the inoculation of the ovarian cancer cells, which suggests that the cancer cells were fully established when the treatments started. These results suggest that the genetic background of the 4T1 cell line or the BALB/c mice are not artificially increasing the activity of our diets. They also show that the anticancer activity of our diets is not limited to a particular type of cancer. As shown in Table 5, a total of 23 mice with different types of metastatic cancers (including TNBC) were treated with diet TB3; the global mean survival was 30.5 ± 2.2 days for untreated mice and 45.4 ± 6.4 days for mice fed diet TB3 (Figure 7 and Table 5).

Our next approach to improve the activity of our diets was to reduce the levels of all AAs by using a low percentage of the protein casein. Proteins allow the sustained liberation and absorption of AAs. After observing that 6% was the lowest percentage of casein required to avoid weight loss in mice (results not shown), we prepared two casein-based diets (TB4 and TB5). Both diets were supplemented with 6% glutamine to keep nitrogen balance. Diet TB4 contained 1% coconut oil, while diet TB5 contained 1% salmon oil and a 5% Leu supplement. Both diets improved the survival of mice with metastatic TNBC, while several cycles of the first-line anticancer drug capecitabine did not improve mice survival under our experimental conditions (Table 6 and Figure 9). Capecitabine actually had a negative effect on mice survival. Possibly, capecitabine did not induce anticancer activity under our experimental conditions, and drug toxicity slightly reduced mice survival. One mouse treated with diet TB4 lived 253 days and a mouse treated with diet TB5 stayed alive for more than one year (448 days).

Our results show for the first time that the survival of mice with metastatic TNBC can be markedly extended by replacing their normal food with an artificial diet. Our most active diets were obtained by manipulating the levels of many AAs simultaneously and by reducing lipid levels to 1%. Importantly, our results were observed in a challenging animal model of metastasis, in which doxorubicin and capecitabine (two first-line treatments for patients with metastatic TNBC) were virtually ineffective. One of our diets (TB3) also induced anticancer activity in mice with other types of metastatic cancers (Figure 7 and Figure 8 and Table 5), therefore suggesting that this anticancer strategy has therapeutic potential for different types of cancer. The clinical translatability of this therapeutic strategy would be straightforward; the normal diet of cancer patients would be temporarily replaced with an artificial diet. Currently, we are evaluating the safety and efficacy of an artificial diet with selective restriction of AAs (6% casein, 5% Gln and 2.5% Leu) and very low levels of lipids (1%) as monotherapy in patients with different types of metastatic cancers.

It is important to note that our artificial diets induced a marked anticancer activity only in some mice with metastatic TNBC. Survival improvements in the rest of the mice were low or non-existent. This is a typical response pattern of immunotherapies, which suggests that our diets may stimulate adaptive immunity to control tumor growth in some mice. In mice with ovarian cancer, however, all mice responded similarly to diet TB3 and lived approximately 2 weeks longer than untreated mice (Figure 7g). It is also important to note that, unlike diets TB1, TB2 and TB4, diets TB3 and TB5 induced a marked weight loss in the animals, which may compromise the safety of these diets in cancer patients. The reason for such a high weight loss cannot be explained by changes in any particular dietary component (see Table 2). For example, the low lipid levels (1%) of diets TB3 and TB5 cannot explain this weight loss, because diets TB2 and TB4 also contain the same percentage of lipids and did not markedly reduce the weight of the animals. Neither the use of casein versus AA mixtures, the amount of Leu, nor the type of lipid can explain the high weight loss observed in animals fed diets TB3 and TB5.

The precise mechanism of action of this therapeutic strategy is unknown and will be difficult to unravel. The first reason is that this anticancer strategy does not use any drug, which makes it challenging to measure interactions with potential cancer drug targets. In addition, because the metabolic environments of cells growing in vitro and in vivo are extremely different, any mechanistic insight obtained in vitro will be difficult to extrapolate to an in vivo situation. For example, in vitro experiments do not consider the fact that liver and muscle proteolysis supplies free AAs to buffer the lack of specific AAs. Proteomics and metabolomics analyses in tumor samples and healthy tissues can provide valuable information on the biological changes elicited by specific artificial diets. Comparing these changes in tumor samples and healthy tissues may help explain why this therapeutic strategy affects cancer cells without causing toxicity in healthy tissues. However, since this therapeutic strategy is based on changing the levels of many nutrients simultaneously, it will be difficult to link biological changes induced by these diets to variations in the levels of specific nutrients. Fortunately, the universally accepted regulatory requirement of benefit over risk does not include mechanism of action as a requisite for approval. In fact, the mechanism of action of numerous approved drugs continues to be unknown or poorly understood (e.g., general anesthetics, lithium, etc. [71,72]). In our opinion, the artificial diets probably create unfavorable metabolic environments for the proliferation and survival of cancer cells. As discussed previously, the DNA aberrations that provide cancer cells with a survival advantage under a normal metabolic environment may become a liability under the new metabolic environments created with our artificial diets. These unfavorable environments can be created with different diets, and these new environments may be toxic to cancer cells with different sets of mutations [64]. This would explain why diets with different compositions are active in a particular type of cancer (e.g., diets TB1-TB5 are active in TNBC), and why the same diet is active in different types of cancer (e.g., diet TB3 is active in TNBC, melanoma, colon cancer, lung cancer and ovarian cancer).

Several possible mechanisms may help to explain why diets with restrictions of AAs and lipids induce selective toxicity towards cancer cells. Unlike normal cells, cancer cells may have mutations and other DNA alterations in the metabolic pathways involved in the synthesis of NEAAs and may be unable to obtain sufficient levels if they are eliminated from the diet [64]. Cancer cells may also have a higher dependency on certain NEAAs such as Cys. Cancer cells are known to produce high levels of reactive oxygen species (ROS) such as hydrogen peroxide, and Cys is necessary to generate glutathione (Glu–Cys–Gly), which in turn is crucial for protecting cells from the cytotoxic effects of ROS. Since our diets lack or have very low levels of Cys, they may induce the accumulation of cytotoxic levels of ROS in cancer cells. In addition, cancer cells have higher proliferative demands than most normal cells and need higher levels of EAAs to produce new proteins for the dividing cancer cells. Diets with reduced levels of EAAs would restrict protein synthesis, cell division and tumor growth. As discussed in the Section 1. lipid restriction may also decrease the proliferative capacity of cancer cells by reducing the availability of fatty acids, which are needed to produce lipid membranes for the new cancer cells. Finally, normal cells have functional checkpoints and may move out of the cell cycle into a quiescent state under conditions of nutrient deprivation. Cancer cells, however, usually have mutations and other DNA alterations that may prevent them from arresting the cell cycle under unfavorable conditions. Entering the cell cycle under conditions of nutrient deprivation may cause their death [73].

Although our artificial diets improved the survival of some mice with metastatic TNBC, and were more effective than the anticancer drugs doxorubicin and capecitabine, all mice eventually died. Future research is needed to improve the efficacy of this non-pharmacological strategy. This work shows that the anticancer activity of diets based on AA manipulation is increased when the levels of lipids are drastically reduced. The anticancer activity of our diets may be further increased by manipulating other dietary constituents, such as vitamins and minerals. Eliminating specific micronutrients in a normal diet is complex because they are present in most foods. However, since our artificial diets can be prepared from scratch, any dietary component can be completely eliminated. Our preliminary results indicate that eliminating specific micronutrients from the artificial diets can increase their anticancer activity in mice with metastatic cancers. In addition, although our diets may be clinically useful as monotherapy, future research would be important to evaluate their anticancer activity in combination with the standard drugs used in cancer patients.

## 5. Conclusions

Current treatments for patients with metastatic TNBC are generally ineffective. Our study revealed that the survival of some mice with metastatic TNBC was markedly increased by replacing their normal diet with artificial diets in which the levels of AAs and lipids are strongly manipulated. The anticancer activity of this non-pharmacological strategy was higher than the activity of drugs currently used in the treatment of patients with metastatic TNBC. This anticancer strategy also increased the survival of mice with different types of metastatic cancers. Diets TB3 and TB5 induced a marked weight loss in the animals, which may compromise the safety of these diets in cancer patients. Manipulating AA and lipid levels with artificial diets may be a useful strategy to treat patients with metastatic disease, including patients with TNBC.

## 6. Patents

E. Guillén-Mancina, J.M Calderón-Montaño, J.J. Jiménez-Alonso, V. Jiménez-González, E. Burgos-Morón, and M. López-Lázaro are inventors of a patent related to this work licensed to AMINOVITA, S.L. and University of Seville.

## Figures and Tables

**Table 1:** Amino acid concentrations in the experimental media (mg/L).

Amino Acid (g/L)	M0	M1
Leucine (Leu)	528	528
Isoleucine (Ile)	96	96
Lysine (Lys)	240	240
Valine (Val)	240	240
Phenylalanine (Phe)	192	192
Threonine (Thr)	160	160
Histidine (His)	80	80
Methionine (Met)	48	48
Tryptophan (Trp)	16	16
Glutamine (Gln)	1000	1000
Glycine (Gly)	200	-
Aspartate (Asp)	20	-
Alanine (Ala)	20	-
Arginine (Arg)	100	-
Serine (Ser)	40	-
Cysteine (Cys)	60	-
Asparagine (Asn)	50	-
Proline (Pro)	20	-
Glutamate (Glu)	20	-
Tyrosine (Tyr)	100	-

**Table 2:** Composition of artificial diets (g/100 g diet).

Artificial Diet	TB1	TB2	TB3	TB4	TB5
Dietary Constituents					
Casein	-	-	-	6.00	6.00
Glutamine (Gln)	6.00	6.00	6.00	5.00	5.00
Leucine (Leu)	6.00	6.00	6.00	-	5.00
Methionine (Met)	0.60	0.60	0.17	-	-
Phenylalanine (Phe)	2.16	2.16	0.61	-	-
Histidine (His)	0.85	0.85	0.24	-	-
Lysine (Lys)	2.64	2.64	0.72	-	-
Threonine (Thr)	1.80	1.80	0.56	-	-
Isoleucine (Ile)	1.07	1.07	0.30	-	-
Valine (Val)	2.64	2.64	0.72	-	-
Tryptophan (Trp)	0.24	0.24	0.07	-	-
Cystine (CySS)	-	-	-	-	-
Arginine (Arg)	-	-	-	-	-
Glycine (Gly)	-	-	-	-	-
Serine (Ser)	-	-	-	-	-
Tyrosine (Tyr)	-	-	-	-	-
Alanine (Ala)	-	-	-	-	-
Aspartate (Asp)	-	-	-	-	-
Proline (Pro)	-	-	-	-	-
Asparagine (Asn)	-	-	-	-	-
Glutamate (Glu)	-	-	-	-	-
Olive oil	14.00	1.00	-	-	-
Coconut oil	-	-	1.00	1.00	-
Salmon oil	-	-	-	-	1.00
Choline	0.25	0.25	0.25	0.25	0.25
Vitamin Mix	1.00	1.00	1.00	1.00	1.00
Mineral Mix	3.50	3.50	3.50	3.50	3.50
Sucrose	15.00	15.00	15.00	15.00	15.00
Cellulose	5.00	5.00	5.00	5.00	5.00
Corn starch	37.25	50.25	58.86	63.25	58.25
Total (g or %)	100	100	100	100	100

**Table 3:** Survival of mice with metastatic TNBC treated with diet TB1 or doxorubicin.

Treatment	*n*	Survival Time (Mean ± SEM; Days)	Survival Improvement vs. Control (Days)	*p* Value vs. Control
Control	7	21.6 ± 1.6	-	-
Doxorubicin (0.5 mg/kg)	8	23.6 ± 1.3	+2.1	0.4838
Diet TB1	10	26.5 ± 1.7	+4.9	0.0837

Data are from three independent experiments. The *p*-value was calculated with the Gehan–Breslow–Wilcoxon test. See text for further details.

**Table 4:** Survival of mice with breast cancer treated with diet TB2 or capecitabine.

Treatment	*n*	Survival Time (Mean ± SEM; Days)	Survival Improvement vs. Control (Days)	*p* Value vs. Control
Control	7	30.1 ± 3.5	-	-
Capecitabine (450 mg/kg/day)	4	23.3 ± 4.0	−6.9	0.2349
Diet TB2	7	38.6 ± 9.3	+8.4	0.9496

Data are from two independent experiments. The *p*-value was calculated with the Gehan–Breslow–Wilcoxon test. See text for further details.

**Table 5:** Survival of mice with different type of cancer treated with diet TB3, capecitabine, anti-PD-1 or cisplatin.

		Control	Diet TB3	Capecitabine (450 mg/kg/Day)	Anti-PD-1 (250 µg/Dose)	Cisplatin (5 mg/kg)
Breast cancer	Survival time (Mean ± SEM; days)	26.1 ± 3.2	43.8 ± 16.2	24.2 ± 1.7	-	-
	Survival improvement vs. Control (days)	-	+17.7	−1.9	-	-
	*n*	8	6	10	-	-
Lung cancer	Survival time (Mean ± SEM; days)	26.3 ± 0.7	30.0 ± 0.6	-	23.3 ± 2.0	-
	Survival improvement vs. Control (days)	-	+3.7	-	−3	-
	*n*	3	3	-	3	-
Ovarian cancer	Survival time (Mean ± SEM; days)	48.8 ± 0.8	64.3 ± 1.8	-	-	80.8 ± 2.1
	Survival improvement vs. Control (days)	-	+15.5	-	-	+32.0
	*n*	4	4	-	-	4
Melanoma	Survival time (Mean ± SEM; days)	22.7 ± 01.7	26.8 ± 2.8	-	-	26.3 ± 4.9
	Survival improvement vs. Control (days)	-	+4.1	-	-	+3.7
	*n*	3	3	-	-	4
Colon cancer (i.v.)	Survival time (Mean ± SEM; days)	33.3 ± 4.9	78.0 ± 29.7	36.7 ± 3.2	-	-
	Survival improvement vs. Control (days)	-	+44.7	+3.3	-	-
	*n*	3	3	3	-	-
Colon cancer (i.p.)	Survival time (Mean ± SEM; days)	27.0 ± 4.2	31.3 ± 1.7	28.0 ± 3.5	-	-
	Survival improvement vs. Control (days)	-	+4.3	+1.0	-	-
	*n*	3	3	3	-	-
Total	Survival time (Mean ± SEM; days)	30.5 ± 2.2	45.4 ± 6.4	27.3 ± 1.8	23.3 ± 2.0	57.4 ± 11.2
	Survival improvement vs. Control (days)	-	+14.9	−3.3	−7.2	+26.9
	*n*	24	23	16	3	7

**Table 6:** Survival of mice with metastatic TNBC treated with diets TB4, TB5 or capecitabine.

Treatment	*n*	Survival Time (Mean ± SEM; Days)	Survival Improvement vs. Control (Days)	*p* Value vs. Control
Control	8	26.1 ± 3.2	-	-
Capecitabine (450 mg/kg/day)	10	24.2 ± 1.7	−1.9	1.0000
Diet TB4	8	52.0 ± 28.8	+25.9	0.4256
Diet TB5	9	75.6 ± 46.9	+49.4	0.4678

Data are from two independent experiments. The *p*-value was calculated with the Gehan–Breslow–Wilcoxon test. See text for further details.

## Data Availability

The data presented in this study are available in this article and Supplementary Materials.

## Supplementary Materials

The following supporting information can be downloaded at https://www.mdpi.com/article/10.3390/cancers15051540/s1, Table S1. In vivo cancer models. Table S2. Clinical signs and symptoms considered to sacrifice mice. Table S3. Composition of artificial media. Figure S1. Evaluation of amino acid restriction on breast cancer cells and non-malignant cells. Figure S2. Lung photographs at the time of sacrifice of mice with TNBC treated with capecitabine or diet TB2. Figure S3. Evaluation of amino acid restriction on a panel of human cancer cell lines of different tissue origin. Figure S4. Photographs at the time of sacrifice of mice with different types of metastatic cancers treated with diet TB3. Figure S5. Autopsy images of mice treated with diets TB4 and TB5.
