# Supplementary material for: Artificial Diets with Selective Restriction of Amino Acids and Very Low Levels of Lipids Induce Anticancer Activity in Mice with Metastatic Triple-Negative Breast Cancer
Source: Cancers (Basel). 2023 Feb 28;15(5):1540. doi: 10.3390/cancers15051540 (PMC10000978; doi:10.3390/cancers15051540)
Supplement: Supplementary file 1 [file cancers-15-01540-s001.zip › cancers-2141805-supplementary.pdf]

cancers

MDPI

Artificial diets with selective restriction of amino acids and very low levels of lipids induce anticancer activity in mice with metastatic triple-negative breast cancer

Emilio Guillén-Mancina , Julio José Jiménez-Alonso , José Manuel Calderón-Montaño , Víctor Jiménez-González , Patricia Díaz-Ortega , Estefanía Burgos-Morón , and Miguel López-Lázaro

Table S1. In vivo cancer models.

Cancer model	Metastatic localization	Cell line inoculation	Mice (sex, strain)	Positive control drug	Treatments start day*
Triple negative breast cancer	Pulmonary metastases	100,000 4T1 cells in the tail vein	Female BALB/cAnNRj	Doxorubicin 0.5 mg/kg i.p. once weekly for 4 weeks<br>Capecitabine 450 mg/kg/day in the diet 7/7 on/off schedule	8
Colon cancer	Pulmonary metastases	100,000 CT26.WT cells in the tail vein	Female BALB/cAnNRj	Capecitabine 450 mg/kg/day in the diet 7/7 on/off schedule	4
	Peritoneal dissemination	100,000 CT26.WT cells in the peritoneal cavity			
Ovarian cancer	Peritoneal dissemination	5,000,000 ID8 *Trp53* -/- cells in the peritoneal cavity	Female C57BL/6JRj	Cisplatin 5 mg/kg i.p. once a week for 4 weeks	21
Lung cancer	Pulmonary metastases	2,000,000 LL/2 cells in the tail vein	Female C57BL/6JRj	Anti-PD-1 250 µg i.p. every 4 days (4 total doses)	7
Melanoma	Pulmonary metastases	1,000,000 B16-F10 cells in the tail vein	Female C57BL/6JRj	Cisplatin 5 mg/kg i.p. once a week for 4 weeks	4

* Days since cancer cell inoculation. All treatments (anticancer drugs and artificial diets) were started the same day for each cancer model.

Table S2. Clinical signs and symptoms considered to sacrifice mice.

Lung metastasis models	Peritoneal dissemination models
**Main sign**	**Main sign**
Rapid body weight loss (10-20%) over 24- 48-h together with tachypnoea (abnormally fast breathing) and dyspnoea (difficulty in breathing) maintained for 24 h.	Significant abdominal distension where body girth increases 20% (due to ascites burden) maintained for 72 h.
**Other signs considered for sacrifice**	
Tachypnoea and dyspnoea (without body weight loss); Lack of curiosity / Reluctance to move >2h; Significant mobility problems / Immobility >2h; Marked piloerection and marked abnormal gait/posture; Failure to eat or drink over a 24- to 48-h period; Marked body weight loss over a 48-h period after stabilisation of weight loss caused by the experimental diets; Progressive and marked body weight loss together with any sign of disease, weakness or suffering; Self-inflicted mutilation; Tumours that interfere with locomotion; Incontinence or diarrhoea over a 48-h period; Anaemia (pale feet, tail and ears); Excessive aggressiveness; Seizures.	

Table S3. Composition of artificial media. Concentrations in M0 (complete medium) and M1 (selective AA restricted medium) are shown in mg/L. Both artificial media were prepared from scratch in our laboratory. All solid ingredients were mixed in sterile water and sterilized by filtration through 0.2 μm membrane filters. Media were supplemented with FBS and penicillin/streptomycin.

**Compound**	**M0**	**M1**
Calcium chloride dihydrate	265	265
Magnesium sulfate	98	98
Ferric (III) nitrate	0.1	0.1
Potassium chloride	400	400
Sodium phosphate monobasic	109	109
Sodium chloride	6400	6400
D-glucose	4500	4500
Choline chloride	4	4
D -pantothenic acid hemicalcium salt	4	4
Folic acid	4	4
Nicotinamide	4	4
Pyridoxine hydrochloride	4	4
Thiamine hydrochlorie	4	4
Myo-inositol	7.2	7.2
D-biotin	0.2	0.2
Riboflavin	0.4	0.4
vitamin B12	0.005	0.005
Sodium bicarbonate	3700	3700
L-phenylalanine	192	192
L-histidine	76	76
L-lysine	235	235
L-threonine	160	160
L-Isoleucine	95	95
L-valine	235	235
L-leucine	533	533
L-tryptophan	21	21
L-methionine	53	53
L-glutamine	533	533
L-arginine	134	-
Glycine	90	-
L-alanine	90	-
L-aspartic acid	178	-
L-serine	42	-
L-tyrosine	90	-
L-cystine dihydrochloride	64	-
L-asparagine-1-hydrate	50	-
L-glutamic acid	20	-
L-proline	20	-

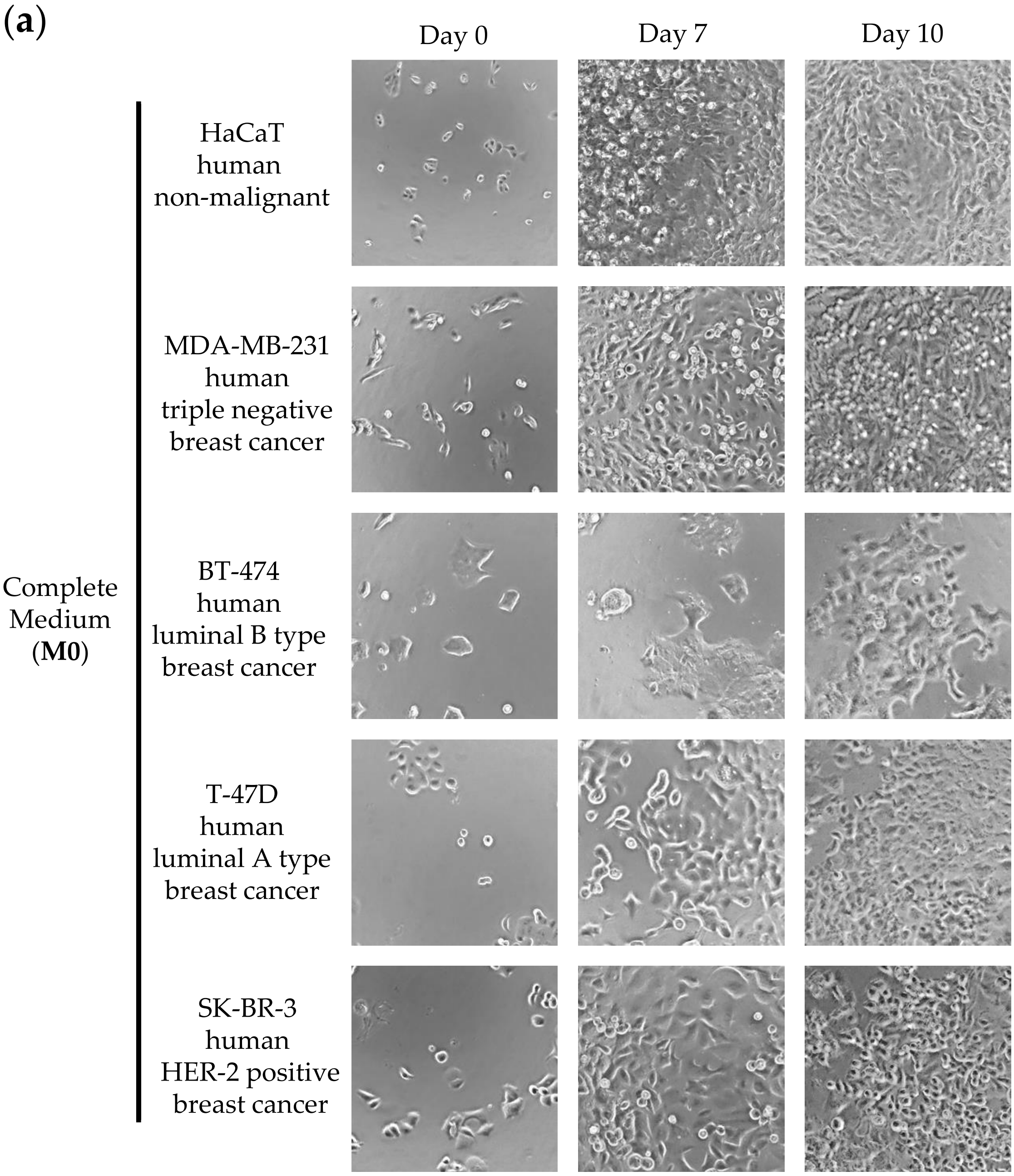

Figure S1. Evaluation of amino acid restriction on breast cancer cells and non-malignant cells. Cells were grown in a complete medium (**M0**) or in a medium lacking 10 AAs (**M1**) for 7 days followed by 3 days of recovery in DMEM medium. T-47D cells were cultured in RPMI 1640. Cells were monitored by microscopic visualization and photographed on days 7 and 10. Representative photographs at 10x magnification are shown. Cell viability was estimated with the resazurin assay on days 7 and 10. The percentage of cell viability is shown at the bottom right of the photographs when it was less than 20%. The detailed composition of M0 (**a**) and M1 (**b**) is shown in Table S3

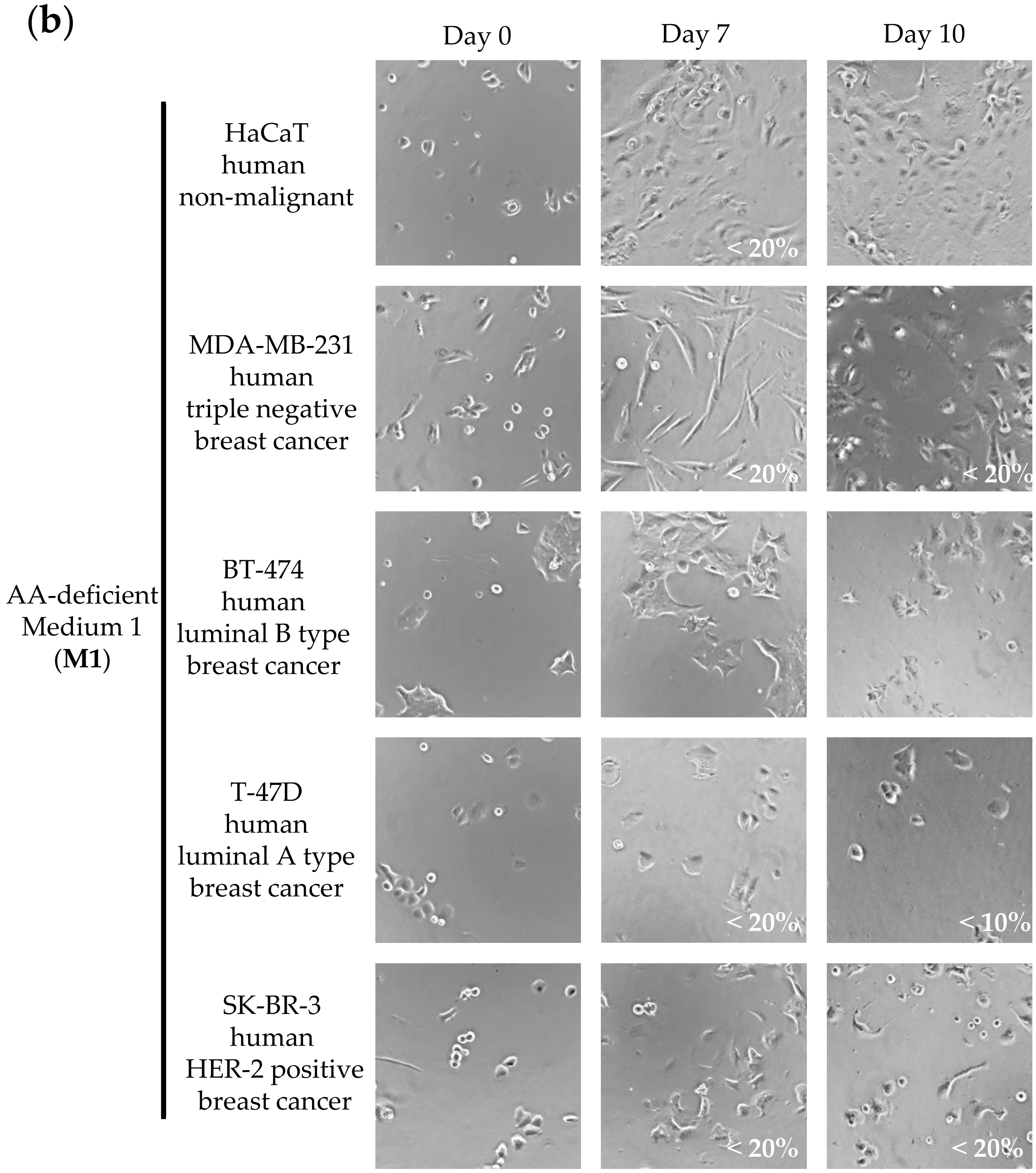

Figure S1. (cont). Evaluation of amino acid restriction on breast cancer cells and non-malignant cells. Cells were grown in a complete medium (**M0**) or in a medium lacking 10 AAs (**M1**) for 7 days followed by 3 days of recovery in DMEM medium. T-47D cells were cultured in RPMI 1640. Cells were monitored by microscopic visualization and photographed on days 7 and 10. Representative photographs at 10x magnification are shown. Cell viability was estimated with the resazurin assay on days 7 and 10. The percentage of cell viability is shown at the bottom right of the photographs when it was less than 20%. The detailed composition of M0 (**a**) and M1 (**b**) is shown in Table S3.

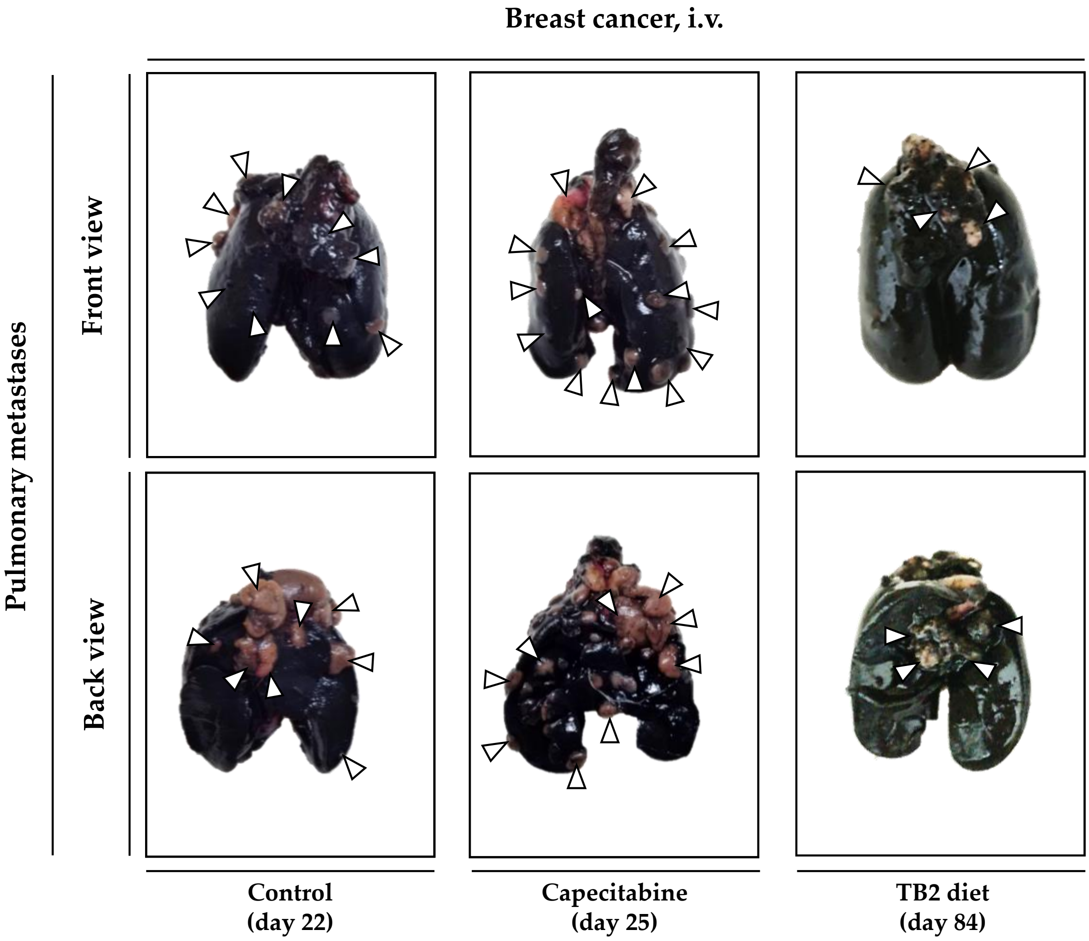

Figure S2. Lung photographs at the time of sacrifice of mice with TNBC treated with capecitabine or diet TB2. In this model, 100 000 4T1 murine breast cancer cells were inoculated in the tail vein of immunocompetent BALB/c mice. After 8 days, mice were treated with capecitabine (450 mg/kg/day, 7/7 schedule, 3 cycles), with diet TB2 (normal diet was replaced by diet TB2 for 4 weeks), or were left untreated (control, normal diet). Mice were euthanized by cervical dislocation when signs of disease progression were apparent. After sacrifice, lungs were excised and stained with India ink (tumors show a white appearance and normal lung parenchyma appears black). Each mouse was sacrificed at different time points, when symptoms of advanced disease were patent. The day of sacrifice is shown in brackets.

(**a**)

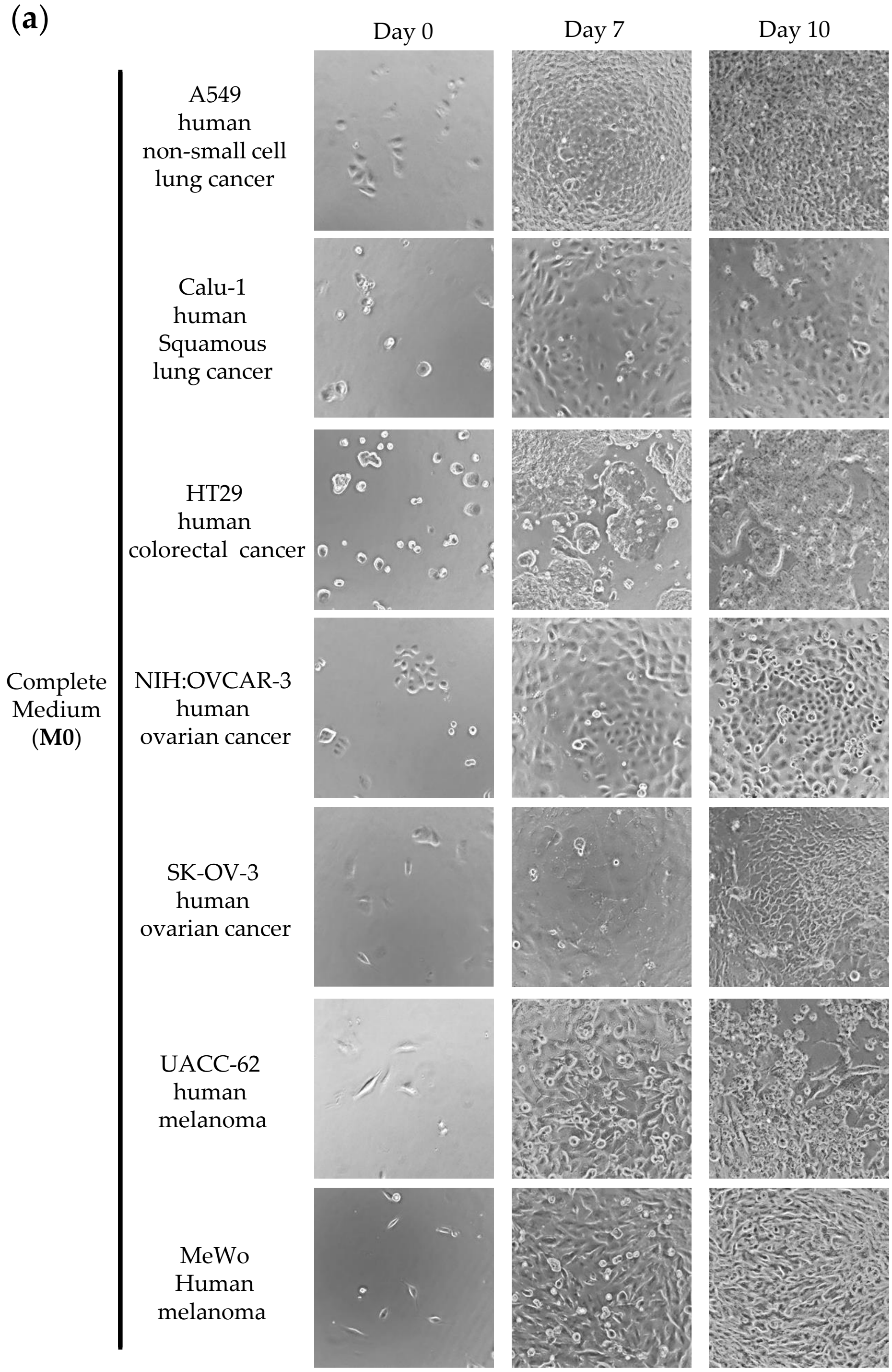

Figure S3. Evaluation of amino acid restriction on a panel of human cancer cell lines of different tissue origin. Cells were grown in a complete medium (**M0**) or in a medium lacking 10 AAs (**M1**) for 7 days followed by 3 days of recovery in DMEM medium. Calu-1, NIH:OVCAR-3 and UACC-62 cells that were cultured in RPMI 1640. Cells were monitored by microscopic visualization and photographed on days 7 and 10. Representative photographs at 10x magnification are shown. Cell viability was estimated with the resazurin assay on days 7 and 10. The percentage of cell viability is shown at the bottom right of the photographs when it was less than 20%. The detailed composition of M0 (**a**) and M1 (**b**) is shown in Table S3.

.

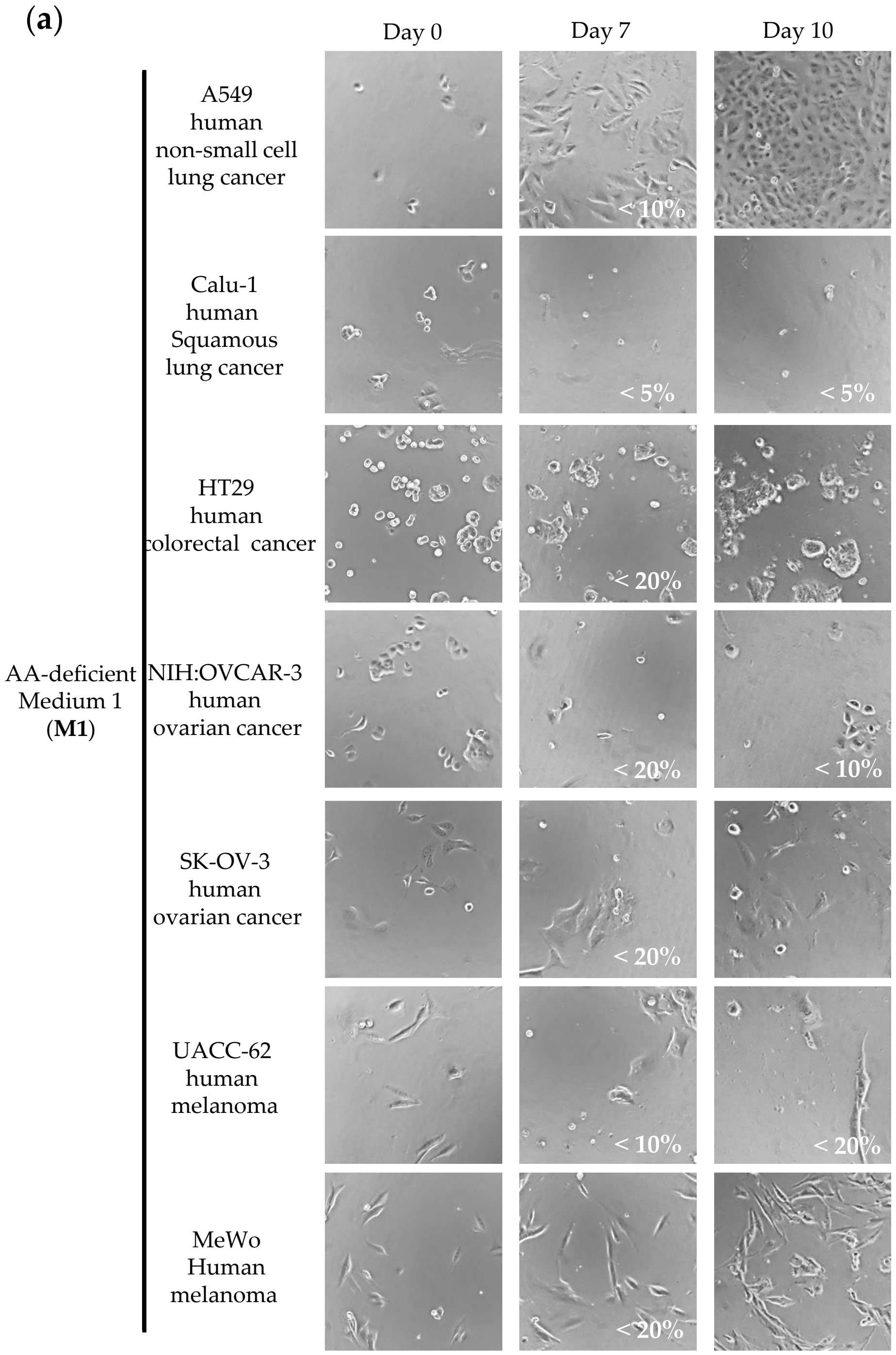

Figure S3. (cont.). Evaluation of amino acid restriction on a panel of human cancer cell lines of different tissue origin. Cells were grown in a complete medium (**M0**) or in a medium lacking 10 AAs (**M1**) for 7 days followed by 3 days of recovery in DMEM medium. Calu-1, NIH:OVCAR-3 and UACC-62 cells that were cultured in RPMI 1640. Cells were monitored by microscopic visualization and photographed on days 7 and 10. Representative photographs at 10x magnification are shown. Cell viability was estimated with the resazurin assay on days 7 and 10. The percentage of cell viability is shown at the bottom right of the photographs when it was less than 20%. The detailed composition of M0 (**a**) and M1 (**b**) is shown in Table S3.

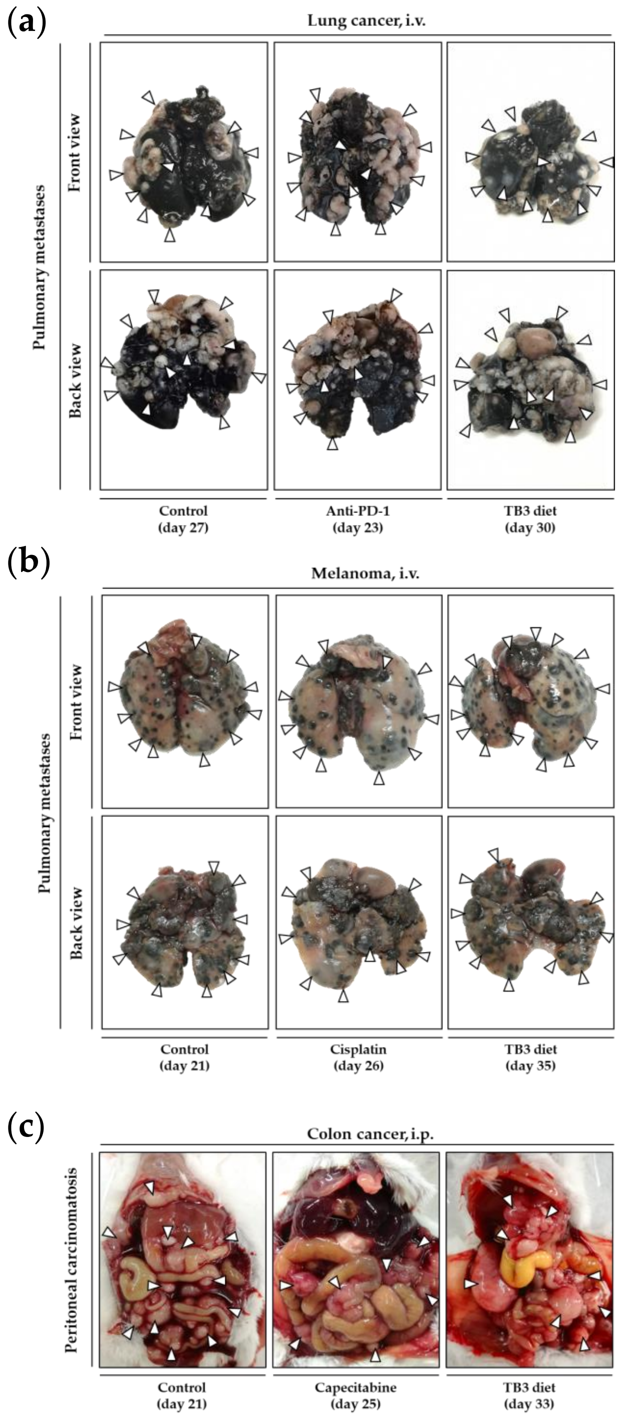

Figure S4. Photographs at the time of sacrifice of mice with different types of metastatic cancers treated with diet TB3. In these models, mice were treated with diet TB3 (normal diet was replaced by diet TB3 for 6 weeks), with a standard anticancer drug, or were left untreated (control, normal diet). In the lung cancer model (**a**), the lungs were excised and stained with India ink (tumors show a white appearance and normal lung parenchyma appears black). In the melanoma model (**b**), representative unstained lung photographs are shown (tumors show a black appearance due to their high production of melanin). In the peritoneal colon cancer model (**c**), representative photographs of the peritoneal cavity are shown. The day of sacrifice is shown in brackets.

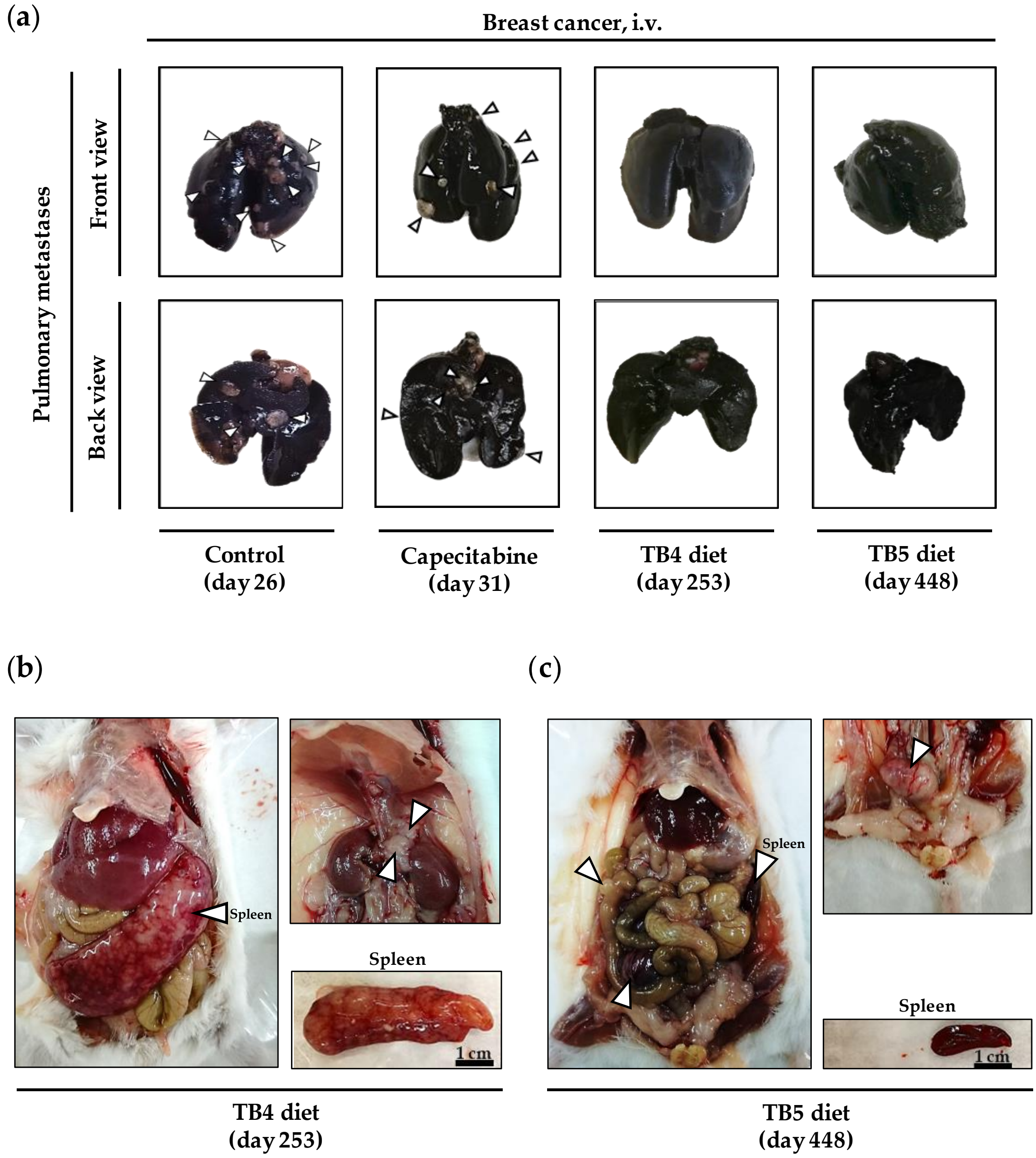

Figure S5. Autopsy images of mice treated with diets TB4 and TB5. In this model, 100 000 4T1 murine breast cancer cells were inoculated in the tail vein of immunocompetent BALB/c mice. After 8 days, mice were treated with capecitabine (450 mg/kg/day, 7/7 schedule, 3 cycles), with diet TB4 or TB5 (normal diet was replaced by TB4 or TB5 for 4 weeks), or were left untreated (control, normal diet). Mice were sacrificed at different time points, when symptoms of advanced disease were patent. The day of sacrifice is shown in brackets. (**a**) Lung photographs at the time of sacrifice of mice with TNBC. After sacrifice, lungs were excised and stained with India ink (tumors show a white appearance and normal lung parenchyma appears black). (**b**) Photographs of the peritoneal cavity and spleen of mice treated with diet TB4 that was sacrificed on day 253. (**c**) Photographs of the peritoneal cavity and spleen of mice treated with diet TB5 that was sacrificed on day 448. See main text for details.
